# Chiral Cu(II)-catalyzed enantioselective β-borylation of α,β-unsaturated nitriles in water

**DOI:** 10.3762/bjoc.11.217

**Published:** 2015-10-27

**Authors:** Lei Zhu, Taku Kitanosono, Pengyu Xu, Shū Kobayashi

**Affiliations:** 1 Department of Chemistry, School of Sciences, The University of Tokyo, Hongo, Bunkyo-ku, Tokyo, Japan

**Keywords:** carbon–boron bond formation, catalytic asymmetric synthesis, chiral copper(II) catalysis, β-hydroxy nitriles

## Abstract

The promising performance of copper(II) complexes was demonstrated for asymmetric boron conjugate addition to α,β-unsaturated nitriles in water. The catalyst system, which consisted of Cu(OAc)_2_ and a chiral 2,2′-bipyridine ligand, enabled β-borylation and chiral induction in water. Subsequent protonation, which was accelerated in aqueous medium, led to high activity of this asymmetric catalysis. Both solid and liquid substrates were suitable despite being insoluble in water.

## Introduction

In recent years, optically active organoboranes have attracted considerable attraction as versatile synthons for the synthesis of biologically interesting compounds and of other materials. In particular, compounds with a nitrile group in the β-position with respect to the boron moiety represent an important subset of organoboron intermediates because these compounds contain two functional groups. Their C–B linkage can be transformed into C–O, C–N, as well as into C–C bonds, while retaining stereogenic centers [1–4]. The nitrile group can be transformed into a range of functional groups, such as amides [5], carboxylic acids [6], aldehydes [7], esters [8], alcohols [9], and amines [10]. Enantioselective boron conjugate addition to α,β-unsaturated nitriles provides one of the most efficient routes to chiral β-boryl nitriles. Several straightforward methods have been developed that rely on chiral Cu(I) complexes with air-sensitive phosphine ligands [11–15]. In contrast, Cu(II)-based catalysis, which has been reported recently for asymmetric boron conjugate addition, is characterized by the effective and thermodynamically stable catalysis in water. Furthermore, a broad range of α,β-unsaturated acceptors, including one example of an α,β-unsaturated nitrile, are applicable, and the reactions, which exhibit extremely high TOF values, can be performed easily without requiring the preparation of an array of chiral ligands [1,16–19]. Rapid protonation in water subsequent to β-borylation would liberate the desired adducts almost instantaneously. In addition to the synthetic utility of enantiomerically enriched β-boryl nitriles, Cu(II)-based activation of α,β-unsaturated nitriles in water is mechanistically curious. In previous reports, homogeneous catalysts composed of Cu(OAc)_2_ were found to be more effective than insoluble Cu(OH)_2_-based catalysts in the asymmetric β-borylation of α,β-unsaturated nitriles in water [1,19]. Herein, we describe the Cu(II)-catalyzed asymmetric boron conjugate addition of α,β-unsaturated nitriles in water.

## Results and Discussion

**Scheme 1:**
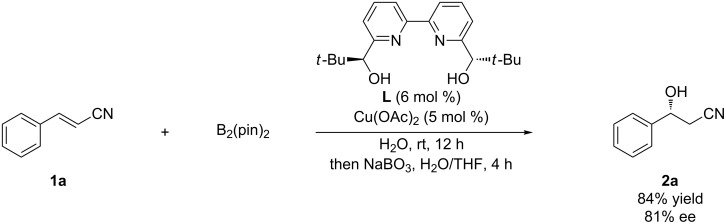
Standard reaction conditions.

At the outset, an aqueous solution of a chiral Cu(II) complex was formed by vigorous stirring of Cu(OAc)_2_ with chiral 2,2′-bipyridine ligand **L** for 1 h. After successive addition of cinnamonitrile (**1a**) and bis(pinacolato)diboron, the resulting mixture was stirred at room temperature for 12 h. Subsequent oxidation by treatment with NaBO_3_ was conducted to determine the enantioselectivity. The desired β-hydroxynitrile **2a** was obtained in 84% yield with 81% ee (Scheme 1), which is consistent with the outcome obtained when the reaction was performed at 5 °C (86% yield, 82% ee [1]). The reactions proceeded smoothly despite the fact that both solid substrates were almost completely insoluble in water. Notably, the desired β-borylated product was isolated as the β-hydroxynitrile after subsequent oxidation, with complete retention of the expected stereochemistry. β-Hydroxynitriles, which are conventionally synthesized by asymmetric addition of acetonitrile to aldehydes [6,20] or by lipase- or nitrilase-catalyzed kinetic resolution of racemic β-hydroxynitriles [21–22], are fascinating candidates for the development of many synthetically feasible derivatives.

When studying these systems, it is important to understand how the conformation of the substrate geometry correlates with its reactivity and enantioselectivity in water. The conjugated system tends to undergo Lewis acid assisted isomerization in water [18]. With three mixtures of **1b** with different *E*/*Z* ratios in hand, their activity toward boron conjugate addition and the sense of stereoselection were examined under the optimal conditions (Table 1). After stirring the reaction mixture for 12 h and subsequent oxidation, β-hydroxynitrile **2b** was obtained in the same yield and with the same enantioselectivity, irrespective of substrate geometry. Notably, the *E*/*Z* geometry of α,β-unsaturated nitrile **1b** did not have an influence on the initial reaction rate, nor was there a difference in the activity or the enantiofacial differentiation between the *E*- and *Z*-isomers.

**Table 1:** Asymmetric β-borylation of **1b** with different configurations.

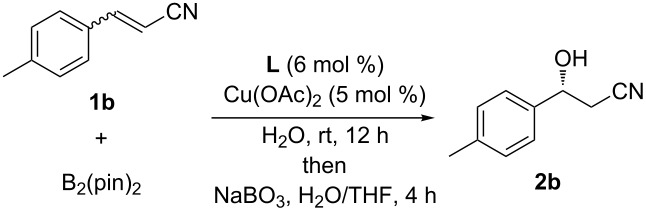			

Entry	*E*/*Z* ratio of **1b**	Yield (%)^a^	ee (%)^b^

1	4.3:1	66	78
2	5.7:1	65	76
3	>99:<1	68	76

^a^Isolated yield. ^b^Determined by chiral HPLC analysis.

The scope of the reaction with respect to α,β-unsaturated nitriles bearing an aromatic ring was then investigated (Table 2). In addition to **1b**, electron-poor α,β-unsaturated nitriles **1c** and **1d** also reacted with the diboron reagent to give the desired products in good to high yields with good to high enantioselectivities. No significant deterioration of chiral induction was observed upon changing the electronic nature of the double bonds. Meanwhile, heterocyclic substrate **1e**, bearing a furan ring, was also tolerated under the reaction conditions and this compound underwent enantioselective β-borylation to afford β-hydroxynitrile **2e** in 87% yield with 87% ee. Remarkably, when β,β-disubstituted α,β-unsaturated nitrile **1f** was employed, the desired β-hydroxynitrile **2f** bearing a quaternary asymmetric carbon center could be successfully produced in 75% yield with 85% ee.

**Table 2:** Scope of the Cu(II)-catalyzed asymmetric borylation with respect to aromatic α,β-unsaturated nitriles.

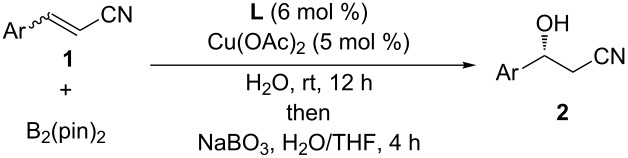				

Entry	Substrate	Product	Yield (%)^a^	ee (%)^b^

1	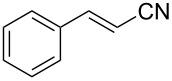 **1a**	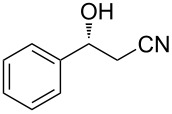 **2a**	84	81
2	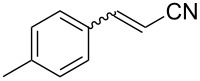 **1b**	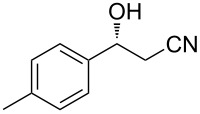 **2b**	66	78
3	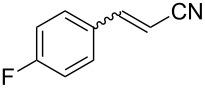 **1c**	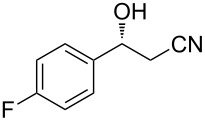 **2c**	92	90
4	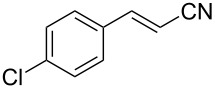 **1d**	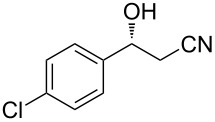 **2d**	75	81
5	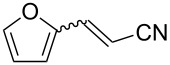 **1e**	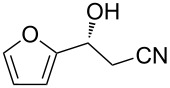 **2e**	87	87
6	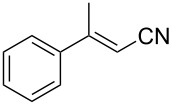 **1f**	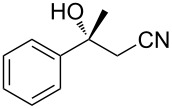 **2f**	75	85

^a^Isolated yield. ^b^Determined by chiral HPLC analysis.

**Scheme 2:**
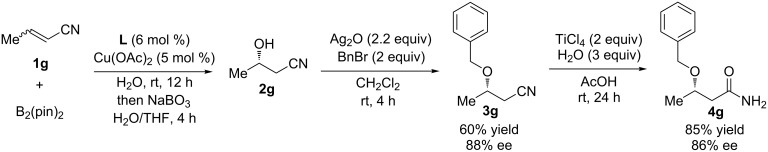
Formation of aliphatic chiral β-hydroxy nitrile **2g** and its subsequent conversion into **4g**.

Finally, to examine the suitability of aliphatic α,β-unsaturated nitriles as substrates for the reaction, the asymmetric β-borylation and subsequent oxidation of crotonitrile (**1g**) were attempted (Scheme 2). The obtained β-hydroxynitrile **2g** was converted into the corresponding benzyl ether **3g** in the presence of Ag_2_O [23], followed by hydrolysis with TiCl_4_ in AcOH [24] without loss of enantiopurity of **3g**. The sense of enantioselection of **3g** was proved to be the same as that of the β-borylated aromatic products shown in Table 2, according to the reported chiral information of β-hydroxyamide **4g** [25]. It is noted that even an aliphatic α,β-unsaturated nitrile worked well under the reaction conditions to afford the desired compound in high yield with high enantioselectivity.

## Conclusion

We have demonstrated that a chiral Cu(II) complex formed with chiral 2,2′-bipyridine ligand **L** constitutes a green and efficient catalyst for asymmetric boron conjugate addition of α,β-unsaturated nitriles in water. Both aromatic and aliphatic α,β-unsaturated nitriles were applicable, and both gave the corresponding chiral β-hydroxynitriles after oxidation with high enantioselectivity. In contrast to well-documented Cu(I) catalytic systems in organic solvents, Cu(II) catalysis in water does not require the use of air-sensitive phosphine ligands or strong bases. Notably, neither the chemical nor physical properties of the α,β-unsaturated nitriles had any influence on either the reactivity or the stereochemical outcome of the reaction; that is, the reaction proceeded well irrespective of whether the sample was solid or liquid, or whether the *E*- or *Z*-isomer was used.

## Supporting Information

File 1 General procedure, analytical data and spectra of all compounds, methods for conversion.
