# Supplementary material for: Chiral Cu(II)-catalyzed enantioselective β-borylation of α,β-unsaturated nitriles in water
Source: Beilstein J Org Chem. 2015 Oct 27;11:2007–11. doi: 10.3762/bjoc.11.217 (PMC4661013; doi:10.3762/bjoc.11.217)
Supplement: File 1 — General procedure, analytical data and spectra of all compounds, methods for conversion. [file Beilstein_J_Org_Chem-11-2007-s001.pdf]

Supporting Information

for

Chiral Cu(II)-catalyzed enantioselective β-borylation of α,β-unsaturated nitriles in water

Lei Zhu, Taku Kitanosono, Pengyu Xu, and Shū Kobayashi*

Address: Department of Chemistry, School of Sciences, The University of Tokyo,
Hongo, Bunkyo-ku, Tokyo, Japan

Email: Shū Kobayashi - shu_kobayashi@chem.s.u-tokyo.ac.jp
* Corresponding author

General procedure, analytical data and spectra of all compounds, methods for conversion

Table of Contents

1. General S1–S5
2. Typical procedure for enantioselective boron conjugate additions S5
3. Optimization of reaction conditions S6
4. Analytical data for oxidized compounds S6–S10
5. References S11
6. ^{1}H NMR and ^{13}C NMR spectra S12–29

1. General

Nuclear magnetic resonance (NMR) spectra were recorded on a JEOL JNM ECX-600 spectrometer, operating at 600 MHz for ^{1}H and 150 MHz for ^{13}C NMR in $CDCl_3$ unless otherwise noted. $CDCl_3$ served as the internal standard (δ = 7.24) for ^{1}H NMR and (δ = 77.0) for ^{13}C NMR. High-performance liquid chromatography was carried out using following apparatures: SHIMADZU LC-20AB (liquid chromatograph), SHIMADZU SPD-20A (UV detector) and SHIMADZU DGU-20A$_3$ (Chromatopac) using Daicel chiralpak® or chiralcel® columns. Preparative thin-layer chromatography (PTLC) was carried out using Wakogel B-5F from Wako Pure Chemical Industries, Ltd. Optical Rotations were measured on a JASCO P1010 polarimeter using a 2 mL cell with 1 dm path length. Data are reported as follows: $[\alpha]_D^T$ (*c* in g/100 mL, solvent). All melting points were determined on a YAZAWA micro melting point BY-1 apparatus and are uncorrected. Deionized water from a MILLIPORE MilliQ machine (Gradient A 10) was used as solvent without further treatment. All organic solvents used were commercially available dry solvents, which were distilled appropriately under an argon atmosphere or were stored over molecular sieves prior to use.

Reagents

α,β-Unsaturated nitriles **1a** and **1g** were purchased from TCI Co., Ltd. and were used directly while **1b–1f** were prepared following the reported method. Analytical data for these compounds are in full agreement with their reported data.

General procedure for the synthesis of α,β-unsaturated nitriles 1b–1f [1]

Under an Ar atmosphere, powdered KOH in dry CH_3CN was heated under reflux and a solution of an aldehyde in dry CH_3CN was then added immediately. After the addition was completed, the stirring was continued for the specified time (vide infra). Afterwards the hot reaction mixture was directly poured onto cracked ice (100 g). The mixture was extracted with 50 mL CH_2Cl_2 three times, and the organic layers

were dried over $MgSO_4$. After filtration and evaporation, the crude product was purified by column chromatography on silica gel.

(*E*)-4-Methylcinnamonitrile (**1b**) [1]

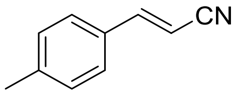

Prepared from 4-methylbenzaldehyde and stirring was continued for 6 min.

White solid (pure *E* isomer): mp 69-71°C; ^{1}H NMR (600 MHz); δ = 7.25-7.29 (m, 3H), 7.12 (d, *J* = 7.5 Hz, 2H), 5.72 (d, *J* = 17.2 Hz, 1H), 2.30 (s, 3H); ^{13}C NMR (150 MHz); δ = 150.5, 141.8, 130.9, 129.8, 127.3, 118.4, 95.1, 21.5.

(*E*)- and (*Z*)-4-Fluorocinnamonitrile (**1c**) [1]

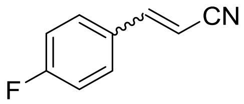

Prepared from 4-fluorobenzaldehyde and stirring was continued for 7 min.

Wet white solid (*E*/*Z* = 5.3/1): ^{1}H NMR (600 MHz); δ (*E*-isomer) = 7.41-7.44 (m, 2H), 7.33 (d, *J* = 16.5 Hz, 1H), 7.06-7.12 (m, 2H), 5.77 (d, *J* = 16.5 Hz, 1H); ^{13}C NMR (150 MHz); δ = 163.5 (d), 149.2, 131.1 (d), 129.3 (d), 117.9, 116.0 (d), 96.1.

(*E*)-4-Chlorocinnamonitrile (**1d**) [1]

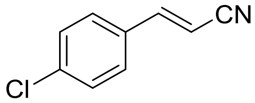

Prepared from 4-chlorobenzaldehyde and stirring was continued for 20 sec.

White solid (pure *E* isomer): mp 85-86°C; ^{1}H NMR (600 MHz); δ = 7.32-7.38 (m, 5H), 5.83 (d, *J* = 16.5 Hz, 1H); ^{13}C NMR (150 MHz); δ = 149.1, 137.3, 131.9, 129.4, 128.5, 117.8, 97.0.

(*E*)- and (*Z*)-2-(2-Furyl)acrylonitrile (**1e**) [1]

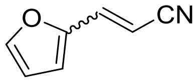

Prepared from furfural and stirring was continued for 3 min.

Yellow liquid (*E*/*Z* = 4/1): ^{1}H NMR (600 MHz); δ (*E*-isomer) = 7.47-7.50 (m, 1H), 7.10-7.18 (m, 1H), 6.60 (d, *J* = 3.4 Hz, 1H), 6.51 (d, *J* = 3.4 Hz, 1H), 5.73 (d, *J* = 16.5 Hz, 1H); ^{13}C NMR (150 MHz); δ =149.8, 145.4, 136.1, 118.2, 115.5, 112.6, 93.4.

(*E*)-3-phenylbut-2-enenitrile (**1f**) [1]

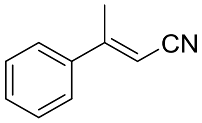

Prepared from acetophenone and stirring was continued for 6 h.

Colourless liquid (pure *E* isomer): ^{1}H NMR (600 MHz); δ = 7.32-7.40 (m, 5H), 5.55 (s, 1H), 2.40 (d, *J* = 1.4 Hz, 3H); ^{13}C NMR (150 MHz); δ =159.8, 138.2, 130.2, 128.8, 125.8, 117.6, 95.5, 20.2.

Bis(pinacolato)diboron [2]

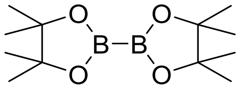

White solid

^{1}H NMR (500 MHz); δ = 1.26 (s, 24H).

^{13}C NMR (125 MHz); δ = 83.5, 25.0.

^{11}B NMR (160 MHz); δ = 30.6 [lit[3] 30.6 ppm].

Metal salts

Anhydrous $Cu(OAc)_2$ was purchased from Kanto Chemical Co., Inc (min 95.0% purity).

Preparation of ligands

Chiral 2,2'-bipyridine **L** was synthesized using protocols described in the literature.

(*S*,*S*)-6,6'-Bis(1-hydroxy-2,2-dimethylpropyl)-2,2'-bipyridine (**L**) [4,5]

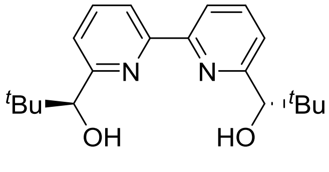

White solid

^{1}H NMR (400 MHz); δ = 8.30 (d, *J* = 8.0 Hz, 2H), 7.79 (dd, *J* = 7.6, 8.0 Hz, 2H), 7.22 (d, *J* = 7.6 Hz, 2H), 4.50-4.43 (m, 2H), 0.98 (s, 18H).

^{13}C NMR (100 MHz); δ = 159.3, 153.8, 136.6, 123.1, 119.6, 80.2, 36.3, 25.9.

HPLC (Dialcel Chiralcel OD, ^{n}hexane/ ^{i}PrOH = 19/1, flow rate 1.0 mL/min); t_R = 40.2 min (*R*, *R*), t_R = 48.7 min (*S*, *S*), t_R = 19.9 min (*meso* isomer). >99.5% *ee*

2. Typical experimental procedure for chiral $Cu(OAc)_2$-catalyzed enantioselective β-borylation to α,β-unsaturated nitriles in water (for compound 2a):

An aqueous solution (2 mL) of $Cu(OAc)_2$ (3.6 mg, 5 mol %) and chiral 2,2'-bipyridine **L** (7.9 mg, 6 mol %) was stirred vigorously for 1 h at room temperature. To the solution, α,β-unsaturated nitrile **1a** (51.7 mg, 0.4 mmol) and $B_2(pin)_2$ (121.8 mg, 0.48 mmol) were successively added. After stirring for 12 h at room temperature, the reaction mixture was extracted with CH_2Cl_2 (20 mL) three times. The combined organic phase was evaporated and the residue was rinsed with THF (3 mL) and H_2O (2 mL). Then an excess amount of $NaBO_3 \cdot 4H_2O$ (488 mg) was added and the mixture was stirred at room temperature for 4 h. The aqueous layer was extracted with CH_2Cl_2 (20 mL) three times, and the combined organic layers were dried over anhydrous $MgSO_4$. After concentration under reduced pressure, the crude mixture was purified by preparative TLC (*n*-hexane/AcOEt 2:1) to afford the desired product **2a** (49.5 mg, 84% yield) as a colourless oil.

3. Optimization of reaction conditions

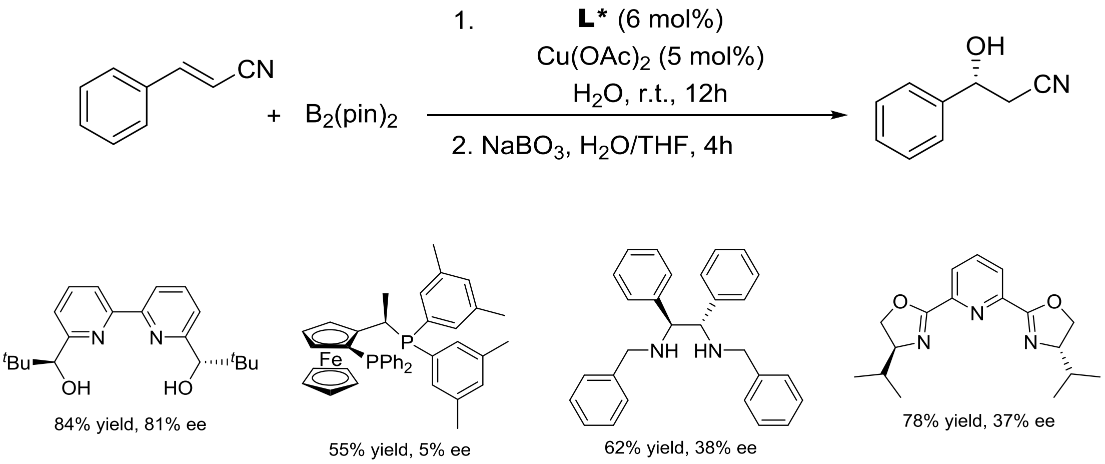

Scheme S1: Screening of chiral ligands.

Table S1: Screening of solvents.

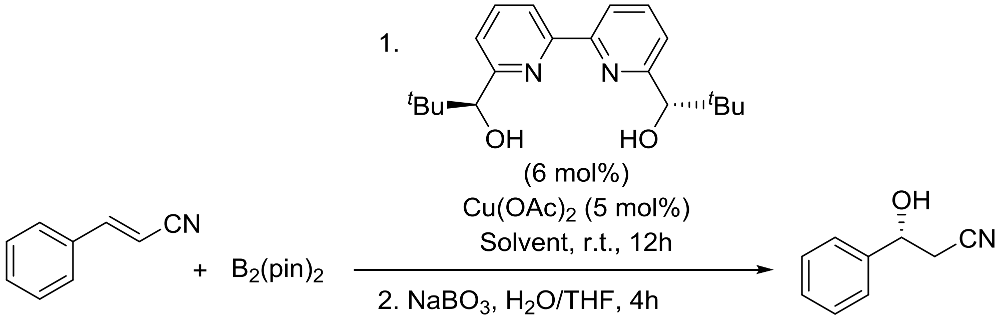

Entry	Solvent	Yield (%)[a]	ee (%)[b]
1	H_2O	84%	81%
2	THF	trace	--
3	CH_2Cl_2	NR	--
4	toluene	NR	--
5	MeOH	25%	36%

4. Analytical data for oxidized compounds

All adducts are literature-known and the obtained analytical data for these compounds are in full agreement with their reported data. The absolute configurations of the optically active compounds were determined by comparison of the order of retention time in the chiral HPLC analyses.

(*R*)-3-Hydroxy-3-phenylpropanenitrile (**2a**) [6]

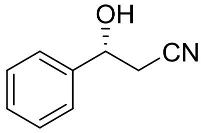

Colourless oil

^{1}H NMR (600 MHz); δ = 2.48 (d, *J* = 3.5 Hz, 1H), 2.75-2.76 (m, 2H), 5.02-5.04 (m, 1H), 7.34-7.39 (m, 5H).

^{13}C NMR (150 MHz); δ = 27.9, 70.2, 117.2, 125.5, 128.9, 129.0, 141.0.

HPLC (Dialcel Chiralcel OJ-H, ^{n}hexane/ ^{i}PrOH = 90/10, flow rate 1.0 mL/min); t_R = 27.8 min (*S*, minor), t_R = 35.0 min (*R*, major).

$[\alpha]_D^{26}$ = + 55.3 (*c* = 0.84, $CHCl_3$).

(*R*)-3-Hydroxy-3-(4-methylphenyl)propanenitrile (**2b**) [6]

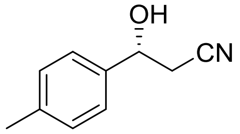

Colourless oil

^{1}H NMR (600 MHz); δ = 2.29 (d, *J* = 3.5 Hz, 1H), 2.34 (s, 3H), 2.70-2.78 (m, 2H), 4.99-5.01 (m, 1H), 7.18-7.19 (d, *J* = 7.8 Hz, 2H), 7.26-7.28 (d, *J* = 8.0 Hz, 2H).

^{13}C NMR (150 MHz); δ = 21,1, 27.9, 70.1, 117.3, 125.4, 129.6, 138.1, 138.8.

HPLC (Dialcel Chiralcel OJ-H, ^{n}hexane/ ^{i}PrOH = 90/10, flow rate 1.0 mL/min); t_R = 21.6 min (*S*, minor), t_R = 25.5 min (*R*, major).

$[\alpha]_D^{26}$ = + 25.6 (*c* = 0.83, $CHCl_3$).

(*R*)-3-(4-Fluorophenyl)-3-hydroxypropanenitrile (**2c**) [6]

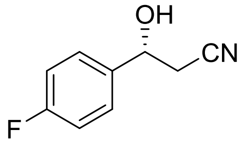

Colourless oil

^{1}H NMR (600 MHz); δ = 2.47 (d, *J* = 3.5 Hz, 1H), 2.72-2.78 (m, 2H), 5.03-5.05 (m, 1H), 7.08 (t, *J* = 8.6 Hz, 2H), 7.37-7.39 (m, 2H).

^{13}C NMR (150 MHz); δ = 28.1, 69.4, 115.8 (d), 117.1, 127.3 (d), 136.9 (d), 162.0 (d).

HPLC (Dialcel Chiralcel OJ-H, ^{n}hexane/ ^{i}PrOH = 90/10, flow rate 1.0 mL/min); t_R = 21.5 min (*S*, minor), t_R = 26.3 min (*R*, major).

$[\alpha]_D^{26}$ = +42.6 (*c* = 0.88, $CHCl_3$).

(*R*)-3-(4-Chlorophenyl)-3-hydroxypropanenitrile (**2d**) [6]

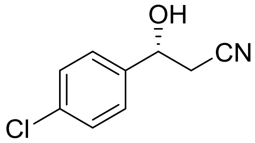

Colourless oil

^{1}H NMR (600 MHz); δ = 2.44 (d, *J* = 3.7 Hz, 1H), 2.74 (t, *J* = 3.4 Hz, 2H), 5.01-5.03 (m, 1H), 7.32-7.37 (m, 4H).

^{13}C NMR (150 MHz); δ = 28.0, 69.5, 116.9, 126.9, 129.1, 134.7, 139.4.

HPLC (Dialcel Chiralcel OJ-H, ^{n}hexane/ ^{i}PrOH = 90/10, flow rate 1.0 mL/min); t_R = 22.5 min (*S*, minor), t_R = 26.6 min (*R*, major).

$[\alpha]_D^{26}$ = + 47.2 (*c* = 0.85, $CHCl_3$).

(*R*)-3-(Furan-2-yl)-3-hydroxypropanenitrile (**2e**) [7]

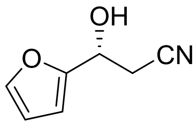

Yellow oil

^{1}H NMR (600 MHz); δ = 2.42 (d, *J* = 5.3 Hz, 1H), 2.89-2.90 (m, 2H), 5.03-5.06 (m, 1H), 6.36-6.40 (m, 2H), 7.39-7.40 (m, 1H).

^{13}C NMR (150 MHz); δ = 24.9, 63.9, 107.5, 110.6, 116.7, 143.0, 152.8.

HPLC (Dialcel Chiralcel OJ-H, ^{n}hexane/ ^{i}PrOH = 90/10, flow rate 1.0 mL/min); t_R = 22.0 min (*S*, minor), t_R = 25.2 min (*R*, major).

$[\alpha]_D^{26}$ = + 37.5 (*c* = 0.82, $CHCl_3$).

(R)-3-hydroxy-3-phenylbutanenitrile (**2f**) [8]

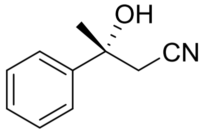

Colourless oil

^{1}H NMR (600 MHz); δ = 1.75 (s, 3H), 2.26 (s, 1H), 2.77-2.84 (m, 2H), 7.29-7.32 (m, 1H), 7.38 (t, J = 7.7 Hz, 2H), 7.46-7.48 (m, 2H).

^{13}C NMR (150 MHz); δ = 29.2, 33.8, 72.5, 117.2, 124.5, 128.1, 128.8, 144.7.

HPLC (Dialcel Chiralcel OJ-H, ^{n}hexane/ ^{i}PrOH = 90/10, flow rate 1.0 mL/min); t_R = 28.3 min (S, minor), t_R = 34.9 min (R, major).

$[\alpha]_D^{26}$ = -25.1 (c = 1.02, $CHCl_3$).

(S)-3-Hydroxybutanenitrile (**2g**) [9]

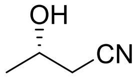

Colourless oil

^{1}H NMR (600 MHz); δ = 1.35 (d, J = 6.2 Hz, 3H), 1.92 (br, 1H), 2.46-2.55 (m, 2H), 4.14-4.17 (m, 1H).

^{13}C NMR (150 MHz); δ = 22.8, 27.5, 64.2, 117.4.

$[\alpha]_D^{26}$ = - 2.0 (c = 0.90, $CHCl_3$).

(S)-3-(Benzyloxy)butanenitrile (**3g**) [10]

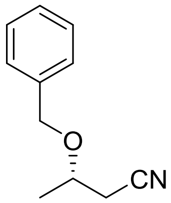

To a mixture of (S)-3-hydroxybutanenitrile **2g** (57.0 mg, 0.67 mmol), benzyl bromide (229 mg, 1.34 mmol) in dichloromethane (4 mL), Ag_2O (348 mg, 1.5 mmol) was added. Then the reaction was stirred at room temperature in dark (foiled) for 4 h. After filtration, solvent was removed by evaporation and residue was purified by PTLC (^{n}hexane/AcOEt = 10/1) to obtain **3g** (70.4 mg, 60% yield) as a colourless oil:

^{1}H NMR (600 MHz); δ = 1.33 (d, *J* = 6.2 Hz, 3H), 2.49-2.57 (m, 2H), 3.81-3.84 (m, 1H), 4.53-4.60 (dd, *J* = 11.7 Hz, 2H), 7.24-7.34 (m, 5H).

^{13}C NMR (150 MHz); δ = 19.7, 25.1, 70.4, 71.0, 117.5, 127,7, 127.9, 128.5, 137.6.

HPLC (Dialcel Chiralpak AD-H, ^{n}hexane/ ^{i}PrOH = 90/10, flow rate 1.0 mL/min); t_R = 11.1 min (*R*, minor), t_R = 13.0 min (*S*, major).

$[\alpha]_D^{26}$ = + 32.1 (*c* = 0.78, $CHCl_3$).

(*S*)-3-(Benzyloxy)butanamide (**4g**) [10]

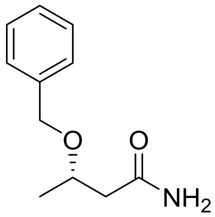

To a solution of (*S*)-3-(benzyloxy)butanenitrile **3g** (52.6 mg, 0.3 mmol) in AcOH (3 mL), $TiCl_4$ (113.8 mg, 0.6 mmol) and H_2O (16.2 mg, 0.9 mmol) were added successively. Then the reaction was stirred at room temperature for 24 h and poured into water. The mixture was extracted by dichloromethane (20 mL) three times and organic phase was combined, dried by $MgSO_4$ and filtered. After removal of solvent by evaporation, the residue was purified by PTLC (^{n}hexane/AcOEt = 4/1) to give **4g** (49.3 mg, 85% yield) as a colourless oil:

^{1}H NMR (600 MHz); δ = 1.29 (d, *J* = 6.2 Hz, 3H), 2.41-2.47 (m, 2H), 3.95-3.98 (m, 1H), 4.46 (d, *J* = 11.4 Hz, 1H), 4.61 (d, *J* = 11.4 Hz, 1H), 5.30 (br, 1H), 6.21 (br, 1H), 7.25-7.34 (m, 5H).

^{13}C NMR (150 MHz); δ = 19.4, 43.3, 70.9, 72.2, 127.7, 127.8, 128.5, 138.0, 173.3.

HPLC (Dialcel Chiralpak AD-H, ^{n}hexane/ ^{i}PrOH = 90/10, flow rate 0.8 mL/min); t_R = 12.4 min (*R*, minor), t_R = 14.2 min (*S*, major).

$[\alpha]_D^{26}$ = + 35.6 (*c* = 0.84, $CHCl_3$).

5. References

1. Dibiase, S. A.; Lipisko, B. A.; Haag, A.; Wolak, R. A.; Gokel, G. W. *J. Org. Chem.* **1979**, *44*, 4640-4649. doi:10.1021/jo00393a037

2. Ishiyama, T.; Murata, M.; Ahiko, T.; Miyaura, N. *Org. Synth.* **2000**, *77*, 176-180. doi:10.15227/orgsyn.077.0176

3. Dewar, M. J. S.; Jones, R. *J. Am. Chem. Soc*. **1967**, *89*, 2408-2410. doi:10.1021/ja00986a029

4. Bolm, C.; Ewald, M.; Felder, M.; Schlingloff, G. *Chem. Ber.* **1992**, *125*, 1169-1190. doi:10.1002/cber.19921250528

5. Ishikawa, S.; Hamada, T.; Manabe, K.; Kobayashi, S. *Synthesis* **2005**, *13*, 2176-2182. doi:10.1055/s-2005-869983

6. Kamila, S.; Zhu, Dunming.; Biehl, E. R. Hua, L. *Org. Lett.* **2006**, *8*, 4429-4431. doi:10.1021/ol061542+

7. Wadhwa, K.; Verkade, J. G. *J. Org. Chem*. **2009**, *74*, 5683-5686. doi:10.1021/ja900814t

8. Suto, Y.; Kumagai, N.; Matsunaga, S.; Kanai, M.; Shibasaki, M. *Org. Lett.* **2003**, *5*, 3147-3150. doi:10.1021/ol035206u

9. Itoh, T.; Mitsukura, K.; Kanphai, W.; Takagi, Y.; Kihara, H.; Tsukube, H. *J. Org. Chem*. **1997**, *62*, 9165-9172. doi:10.1021/jo971288m

10. Ma, D.-Y.; Wang, D.-X.; Pan, J.; Huang, Z.-T.; Wang, M.-X. *J. Org. Chem*. **2008**, *73*, 4087-4091. doi:10.1021/jo800074k

6. ^{1}H NMR, ^{13}C NMR and HPLC spectra

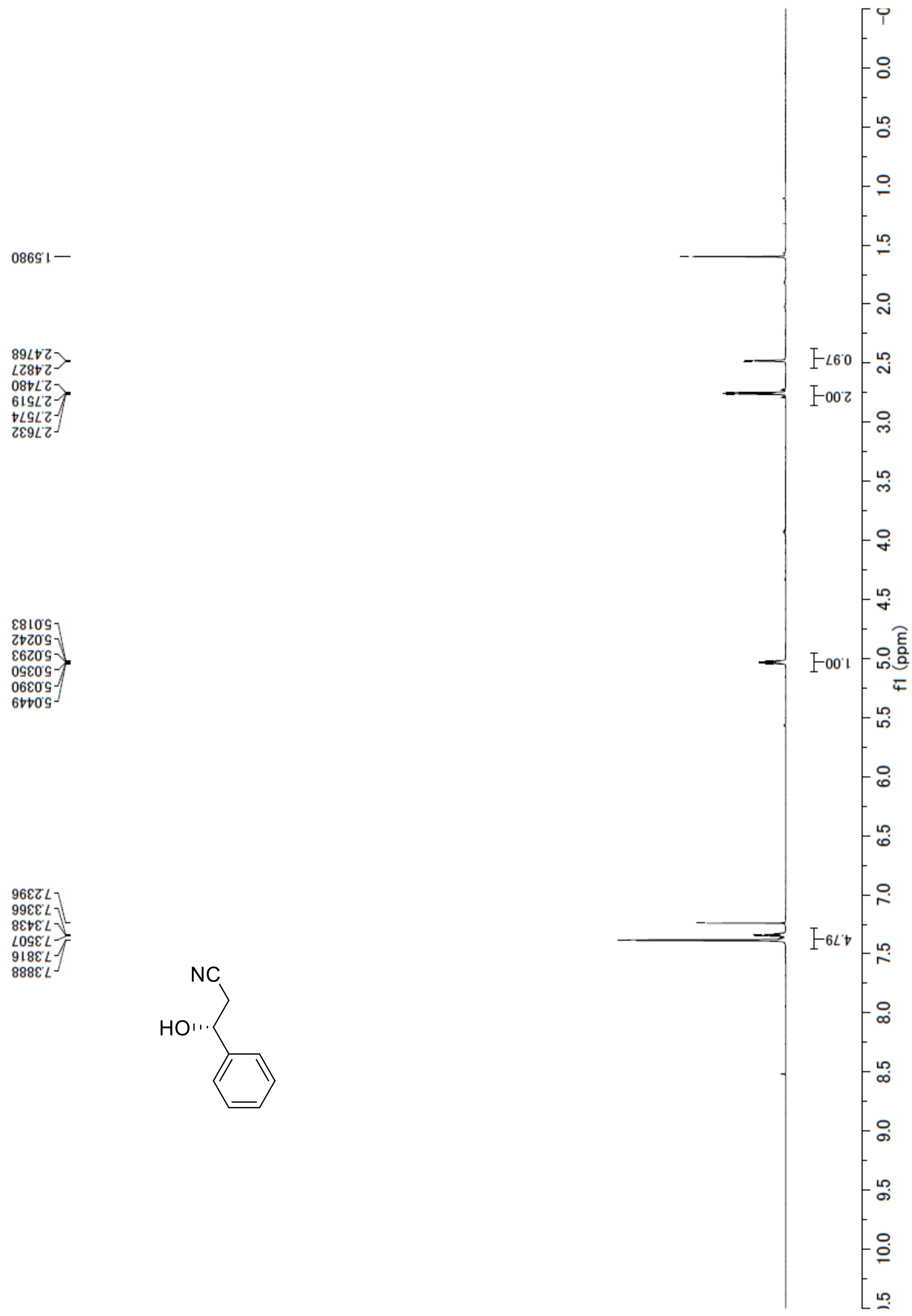

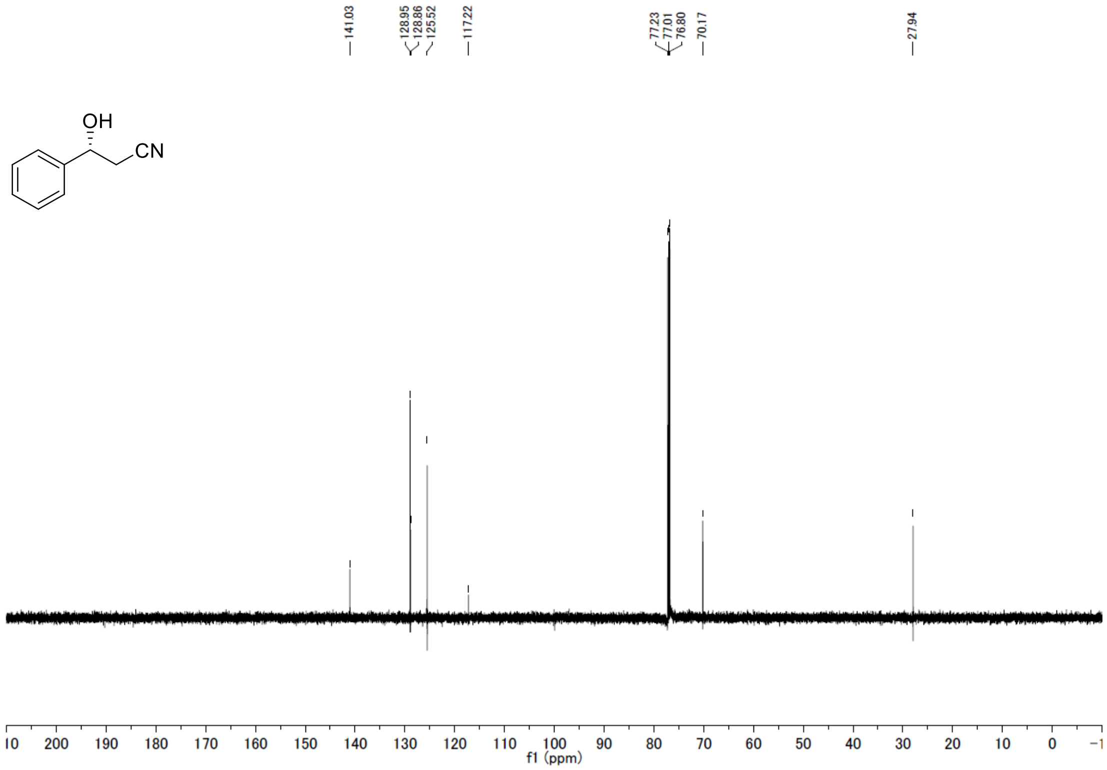

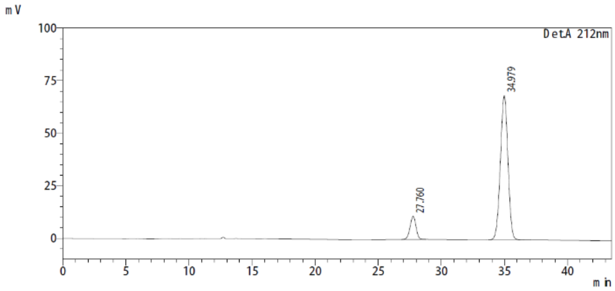

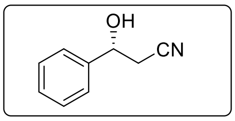

<ピークレポート>
DetA 212nm

ピーク#	保持時間	面積	高さ	濃度	単位	マーク	化合物名
1	27.760	300316	10152	9.494			
2	34.979	2862799	68590	90.506			
Total		3163116	78743				

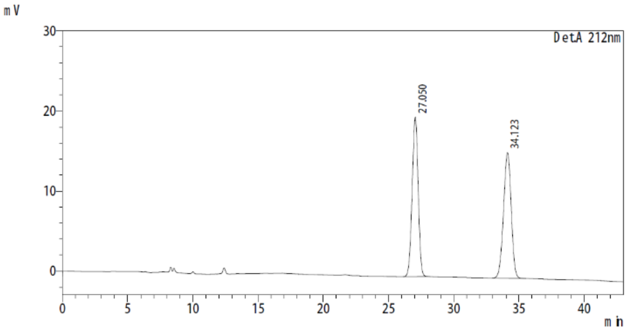

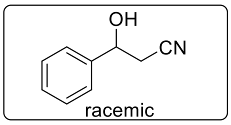

<ピークレポート>
DetA 212nm

ピーク#	保持時間	面積	高さ	濃度	単位	マーク	化合物名
1	27.050	639262	19926	49.926			
2	34.123	641150	15685	50.074			
Total		1280412	35611				

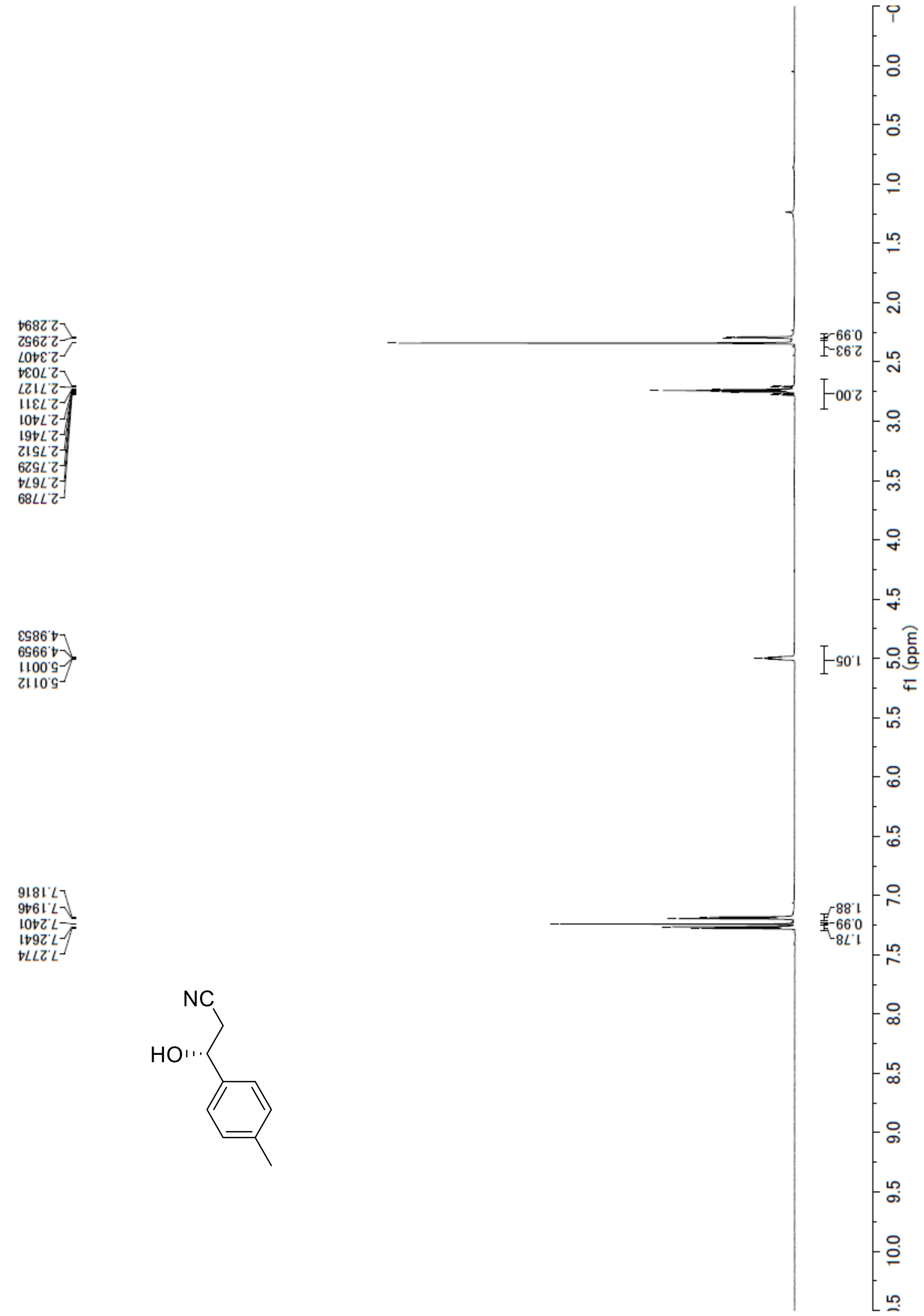

2.2894
2.2952
2.3407
2.7034
2.7127
2.7311
2.7401
2.7461
2.7512
2.7529
2.7674
2.7789
4.9853
4.9959
5.0011
5.0112
7.1816
7.1946
7.2401
7.2641
7.2774
NC
HO
0.99
2.93
2.00
1.05
1.88
0.99
1.78
f1 (ppm)

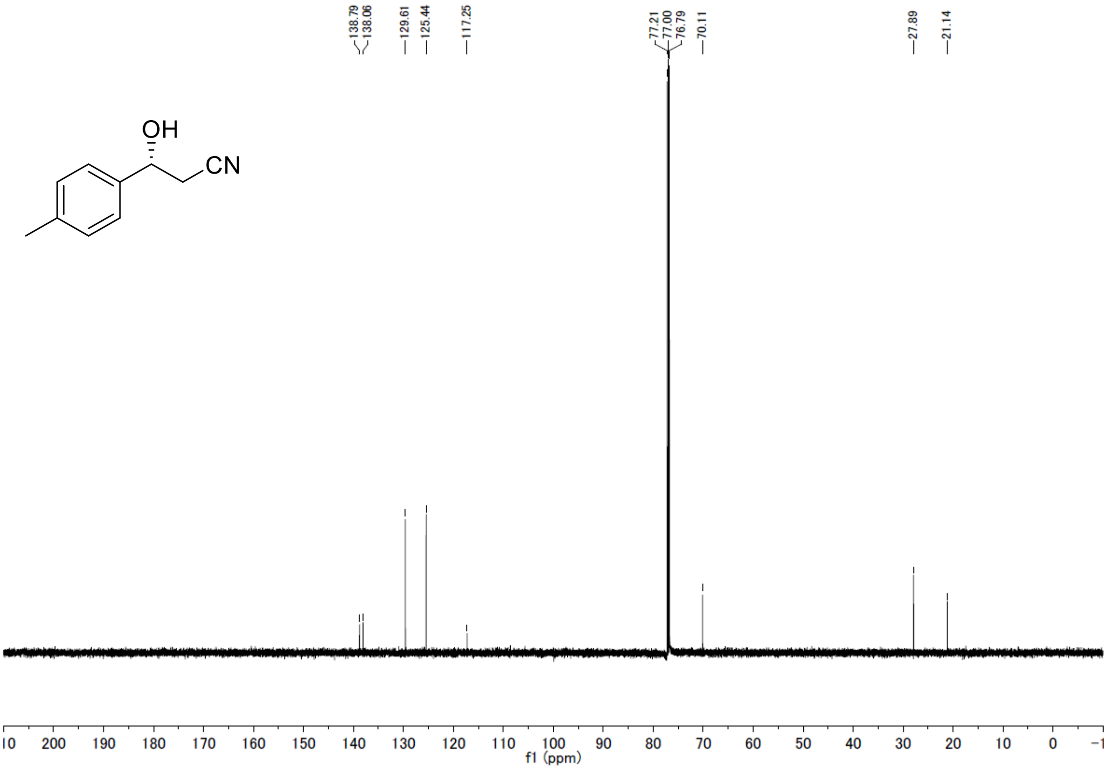

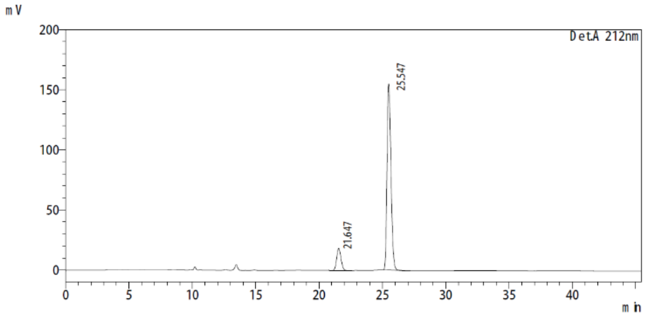

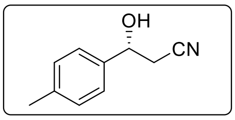

<ピークレポート>
DetA 212nm

ピーク#	保持時間	面積	高さ	濃度	単位	マーク	化合物名
1	21.647	1443498	70581	11.880			
2	25.547	10706660	589410	88.120			
Total		12150157	659991				

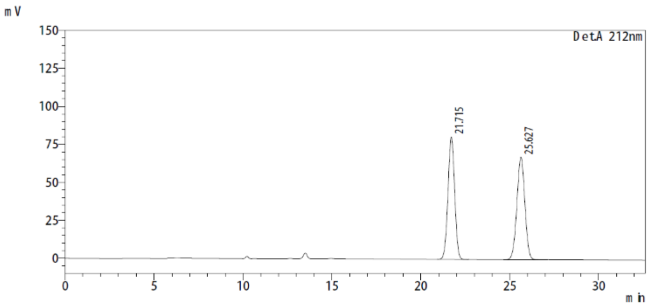

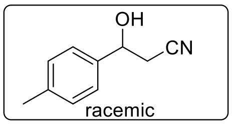

<ピークレポート>
DetA 212nm

ピーク#	保持時間	面積	高さ	濃度	単位	マーク	化合物名
1	21.715	2098988	80494	49.840			
2	25.627	2112472	67416	50.160			
Total		4211460	147910				

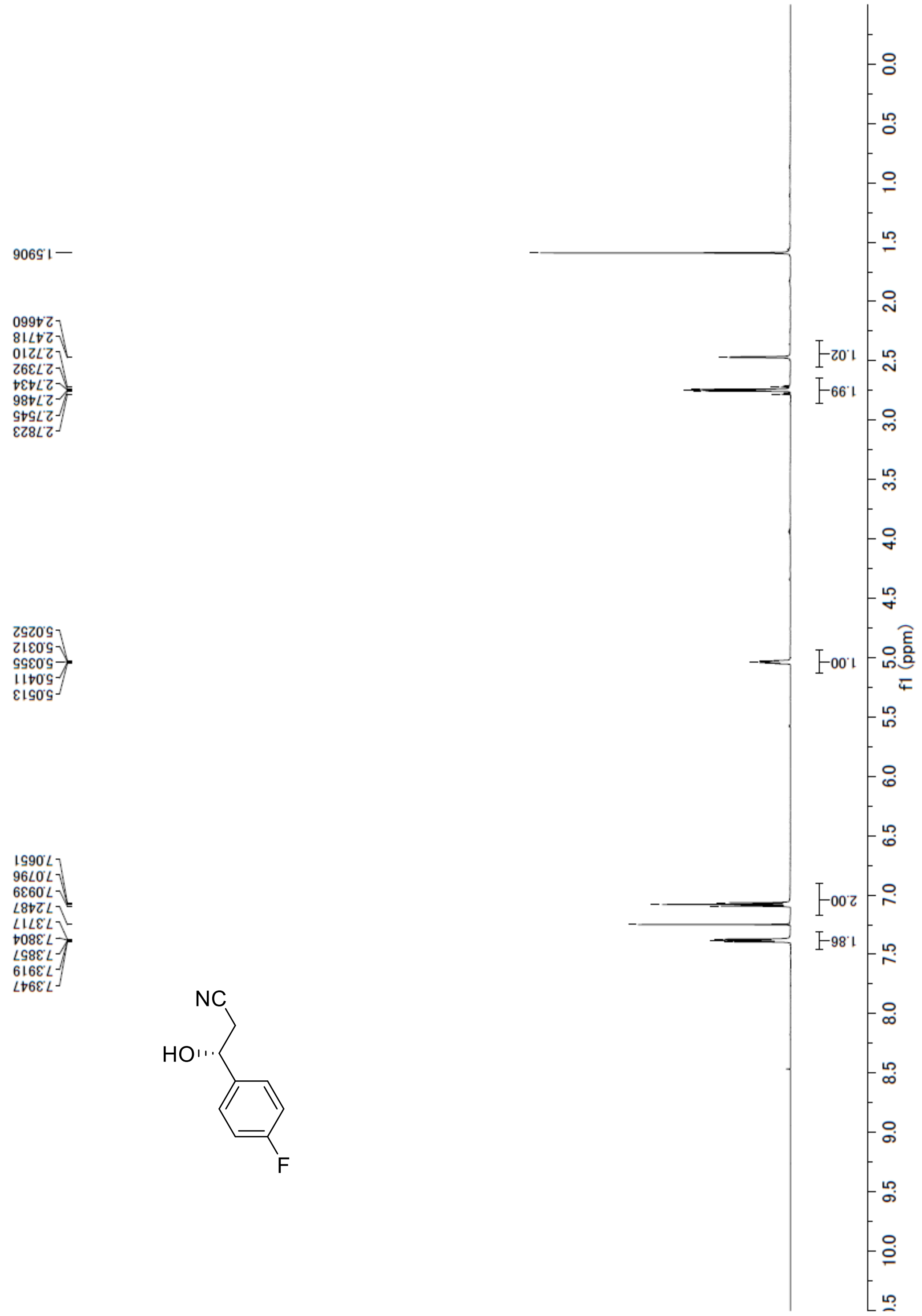
1.5906
2.4660
2.4718
2.7210
2.7392
2.7434
2.7486
2.7545
2.7823
5.0252
5.0312
5.0355
5.0411
5.0513
7.0651
7.0796
7.0939
7.2487
7.3717
7.3804
7.3857
7.3919
7.3947
1.02
1.99
1.00
2.00
1.86
f1 (ppm)
NC
HO
F

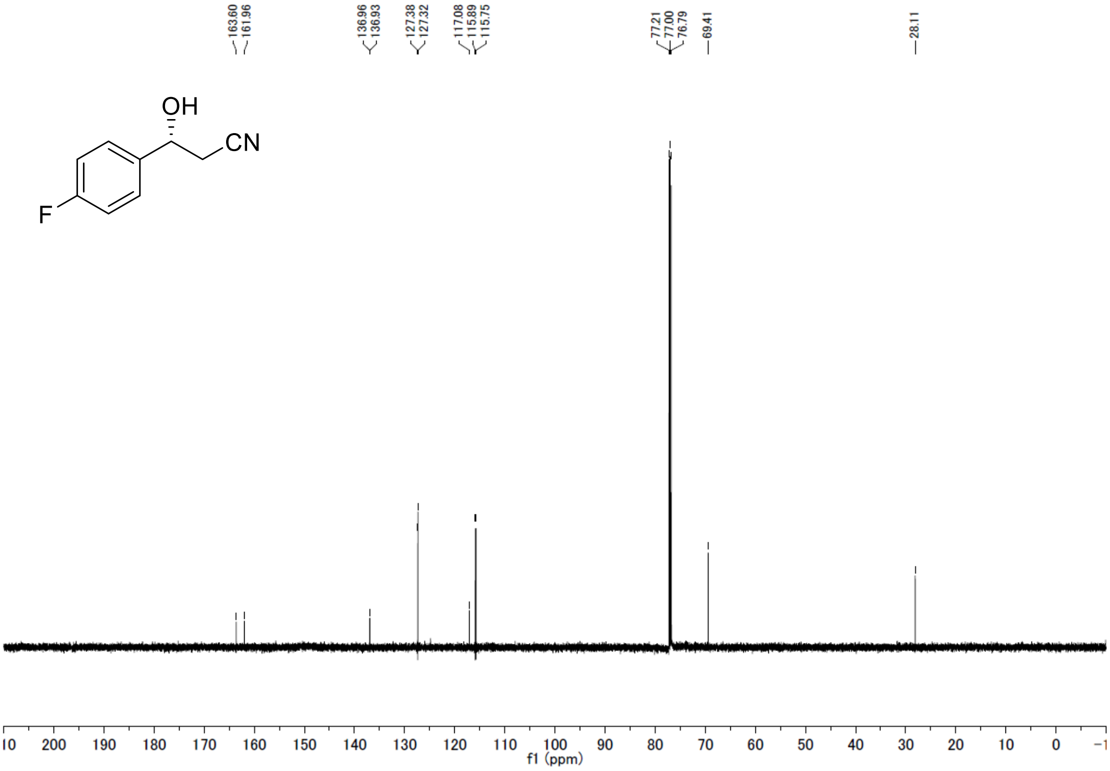

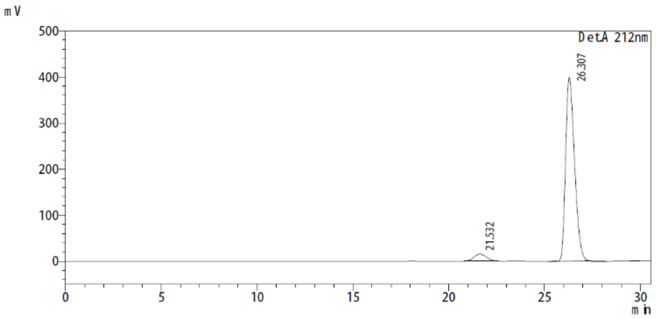

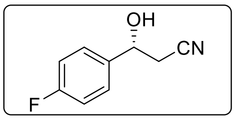

<ピークレポート>

DetA 212nm

ピーク#	保持時間	面積	高さ	濃度	単位	マーク	化合物名
1	21.532	343875	8572	5.145			
2	26.307	6339832	225253	94.855			
Total		6683707	233825				

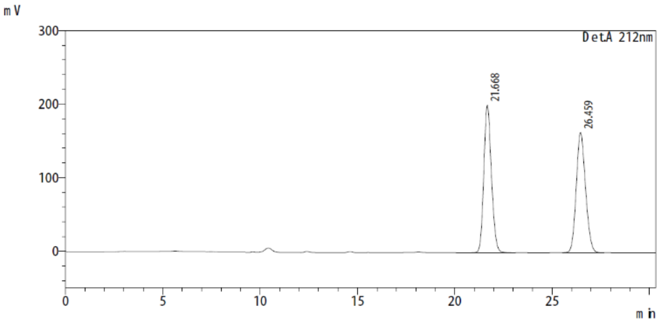

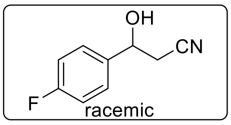

<ピークレポート>

DetA 212nm

ピーク#	保持時間	面積	高さ	濃度	単位	マーク	化合物名
1	21.668	5475976	200716	50.198			
2	26.459	5432698	163419	49.802		M	
Total		10908673	364135				

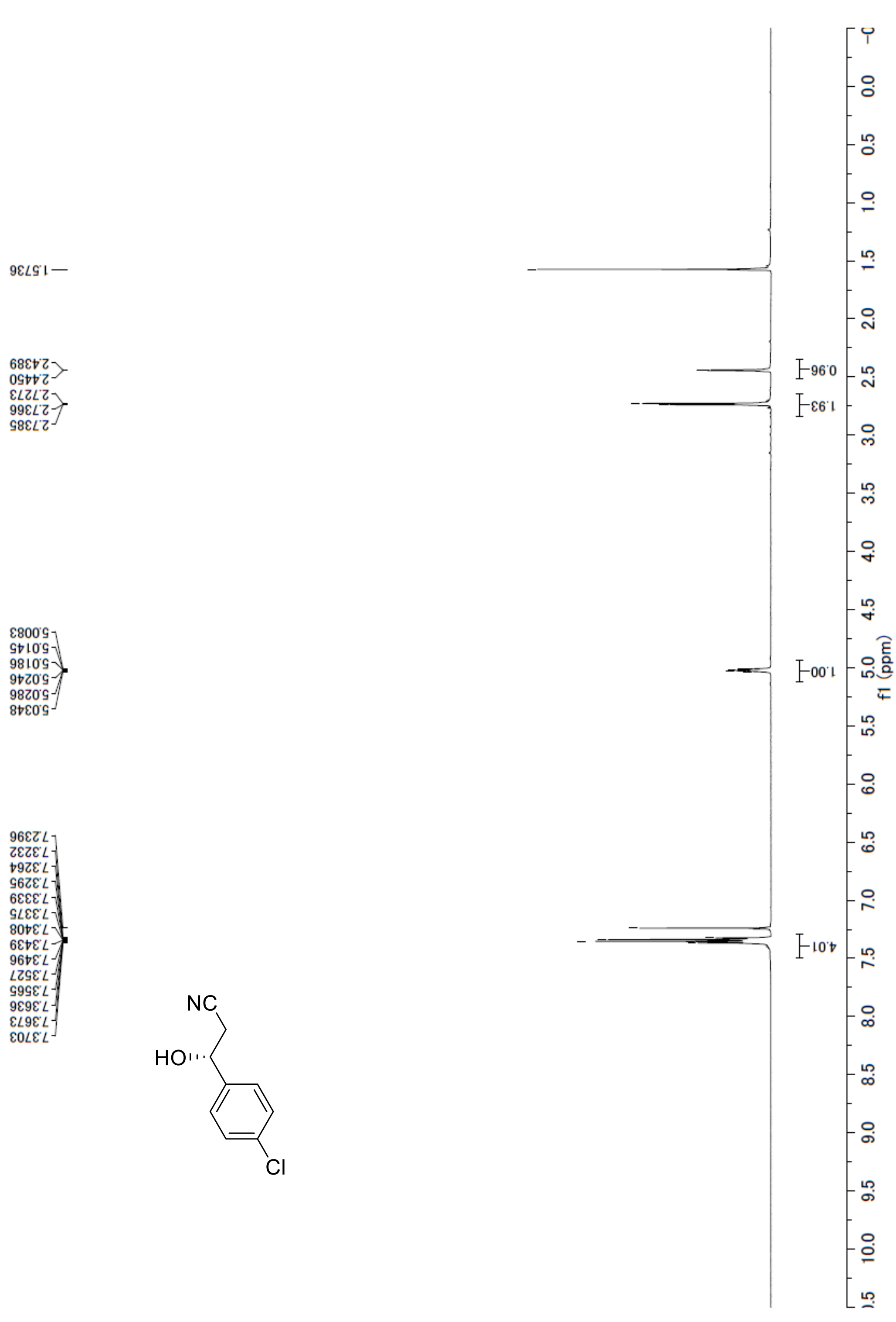
1.5736
2.4389
2.4450
2.7273
2.7366
2.7385
5.0083
5.0145
5.0186
5.0246
5.0286
5.0348
7.2396
7.3232
7.3264
7.3295
7.3339
7.3375
7.3408
7.3439
7.3496
7.3527
7.3565
7.3636
7.3673
7.3703
0.96
1.93
1.00
4.01
f1 (ppm)
NC
HO
Cl

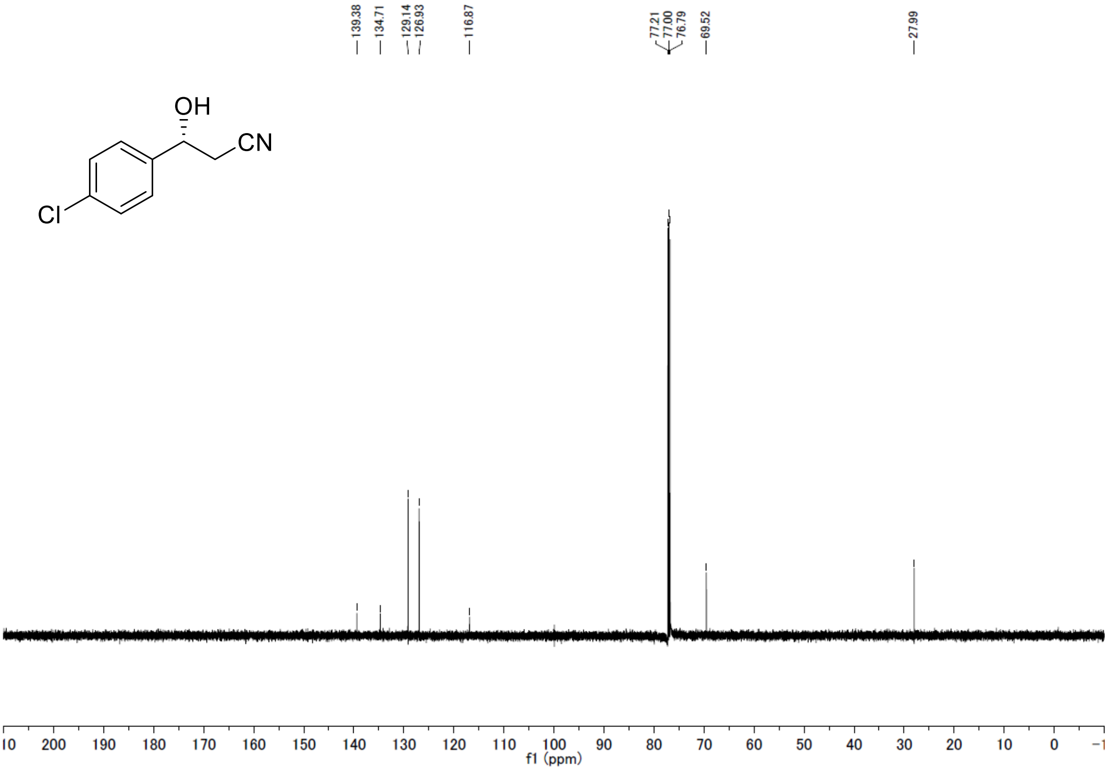

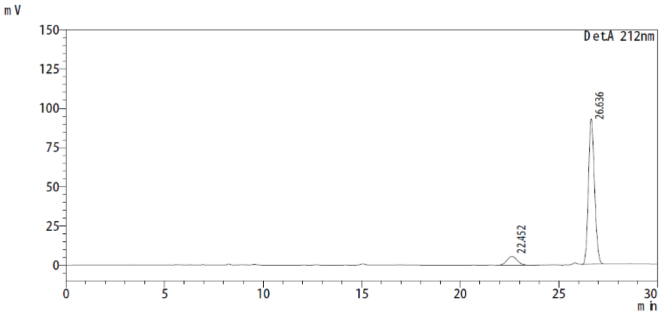

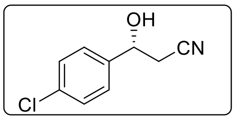

<ピークレポート>
DetA 212nm

ピーク#	保持時間	面積	高さ	濃度	単位	マーク	化合物名
1	22.452	201830	5585	9.263			
2	26.636	1977139	92475	90.737			
Total		2178969	98060				

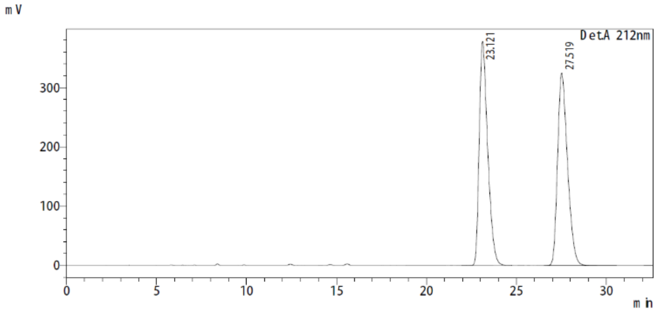

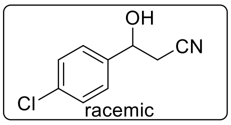

<ピークレポート>
DetA 212nm

ピーク#	保持時間	面積	高さ	濃度	単位	マーク	化合物名
1	23.121	12420516	378154	49.896			
2	27.519	12472114	325235	50.104			
Total		24892630	703389				

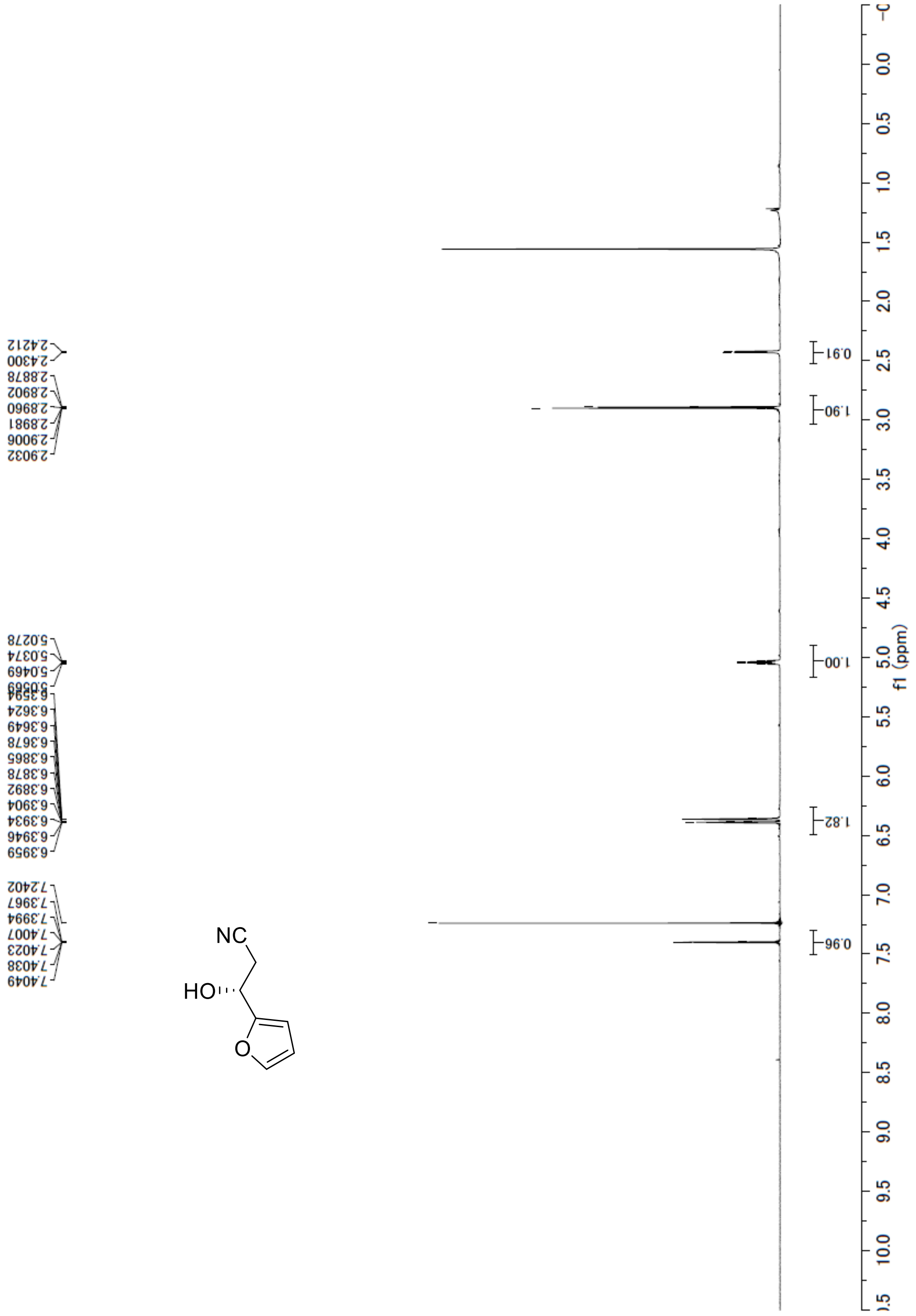

2.4212
2.4300
2.8878
2.8902
2.8960
2.8981
2.9006
2.9032
5.0278
5.0374
5.0469
5.0565
6.3594
6.3624
6.3649
6.3678
6.3865
6.3878
6.3892
6.3904
6.3934
6.3946
6.3959
7.2402
7.3967
7.3994
7.4007
7.4023
7.4038
7.4049
NC
HO
O
0.91
1.90
1.00
1.82
0.96
f1 (ppm)

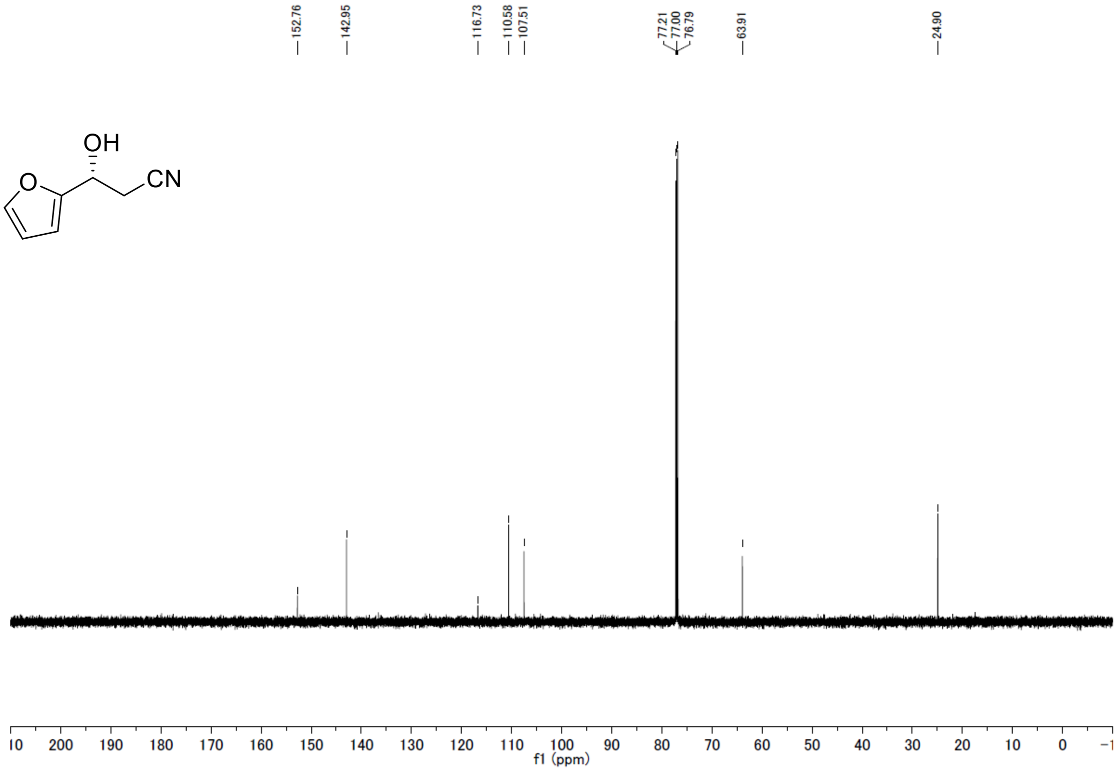

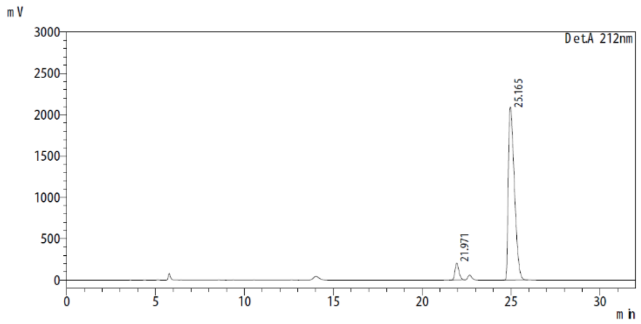

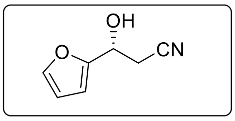

<ピークレポート>
DetA 212nm

ピーク#	保持時間	面積	高さ	濃度	単位	マーク	化合物名
1	21.971	3155900	204163	6.348		M	
2	25.165	46559944	2036162	93.652		M	
Total		49715844	2240325				

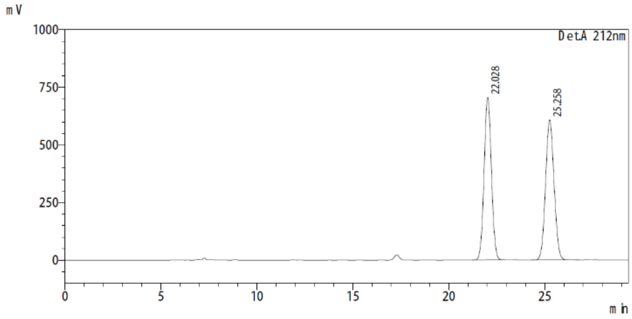

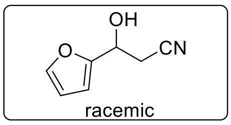

<ピークレポート>
DetA 212nm

ピーク#	保持時間	面積	高さ	濃度	単位	マーク	化合物名
1	22.028	18673636	704569	49.908			
2	25.258	18742455	607778	50.092			
Total		37416090	1312347				

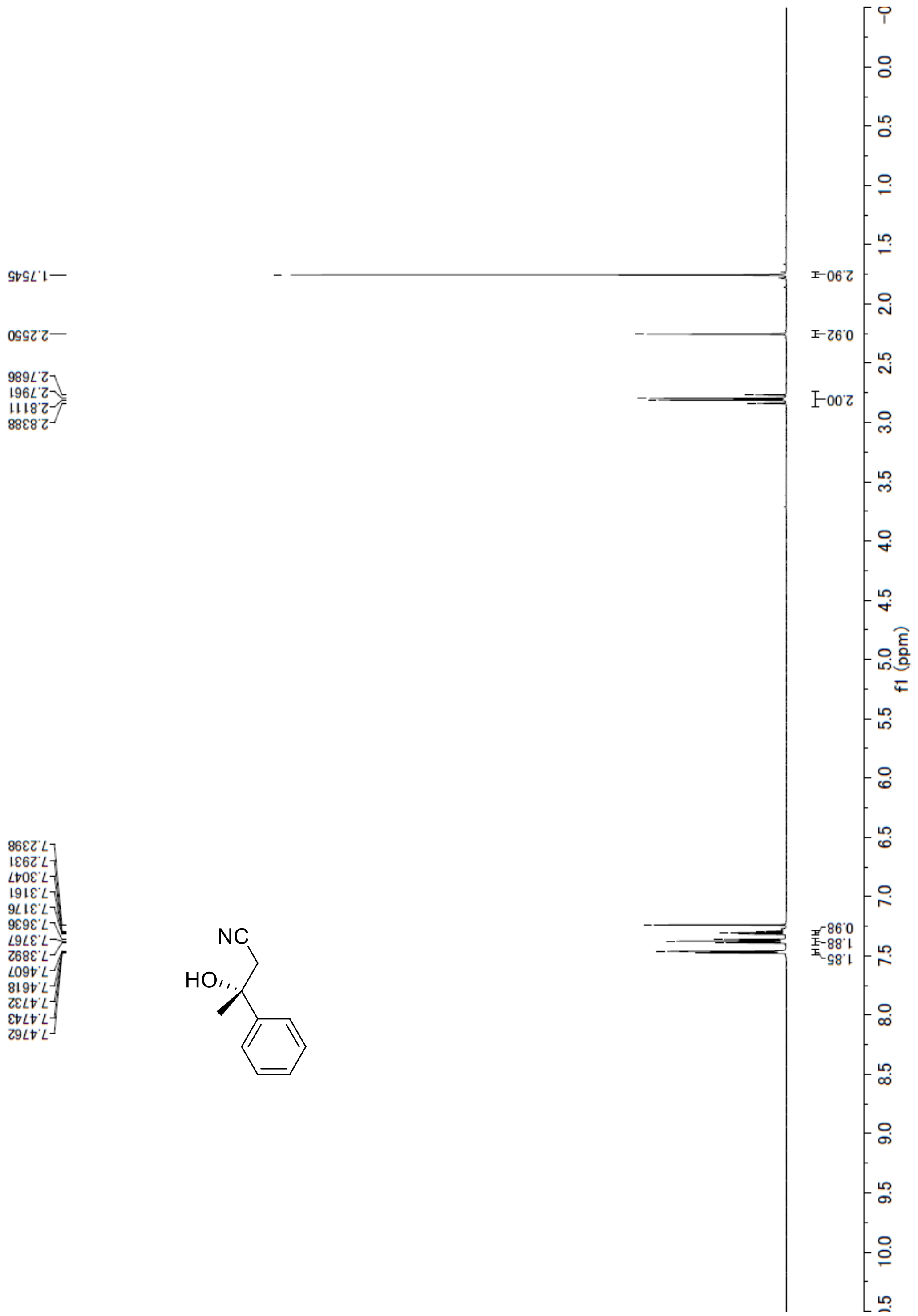

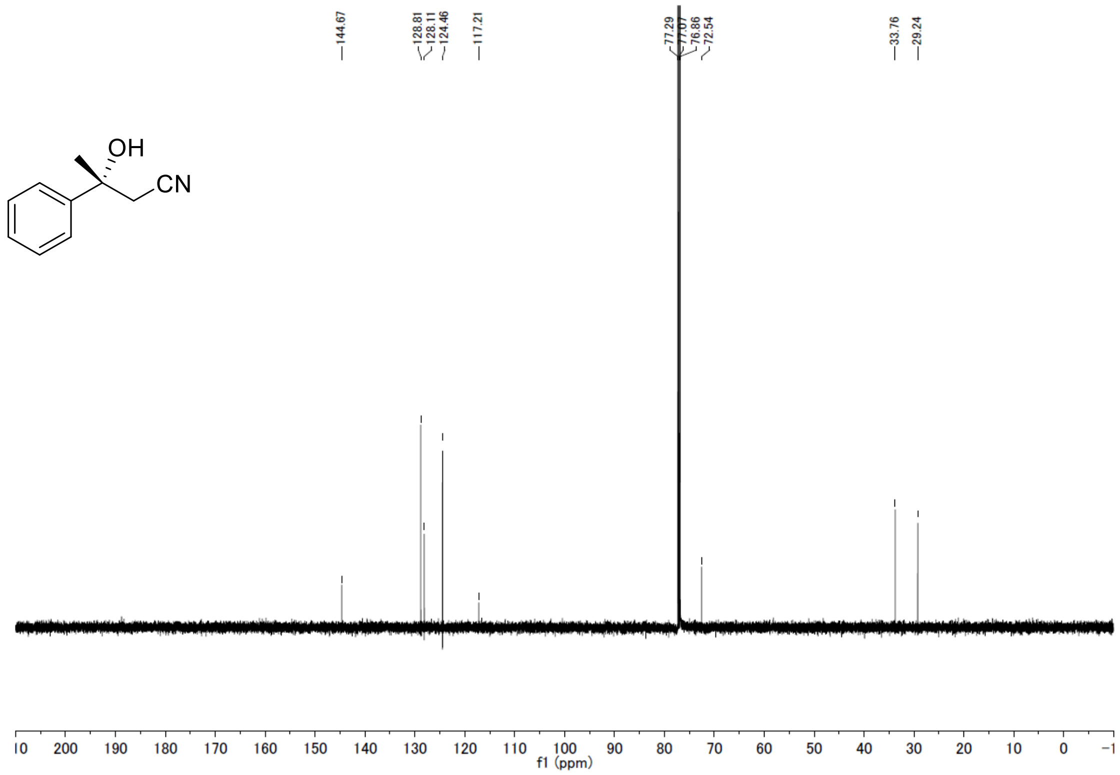

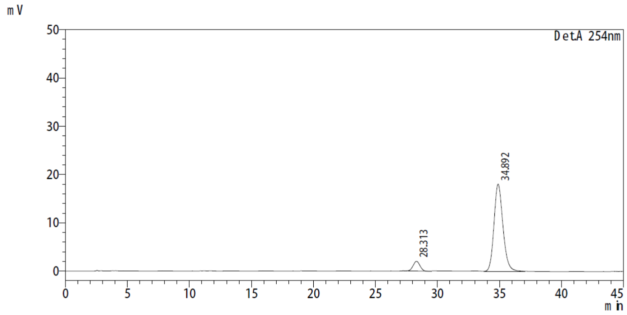

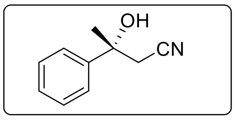

<ピークレポート>
DetA 254nm

ピーク#	保持時間	面積	高さ	濃度	単位	マーク	化合物名
1	28.313	75678	2017	7.694		M	
2	34.892	907887	18063	92.306			
Total		983565	20080				

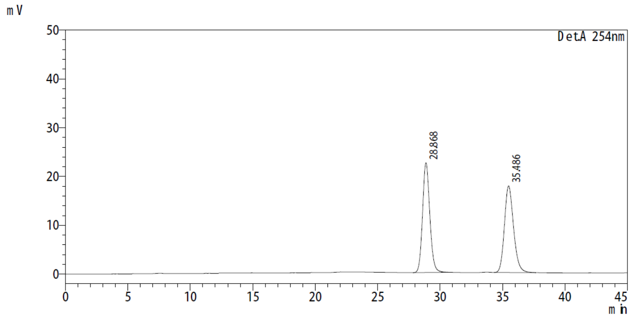

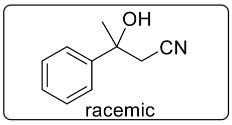

<ピークレポート>
DetA 254nm

ピーク#	保持時間	面積	高さ	濃度	単位	マーク	化合物名
1	28.868	910085	22489	50.190			
2	35.486	903181	17747	49.810			
Total		1813265	40236				

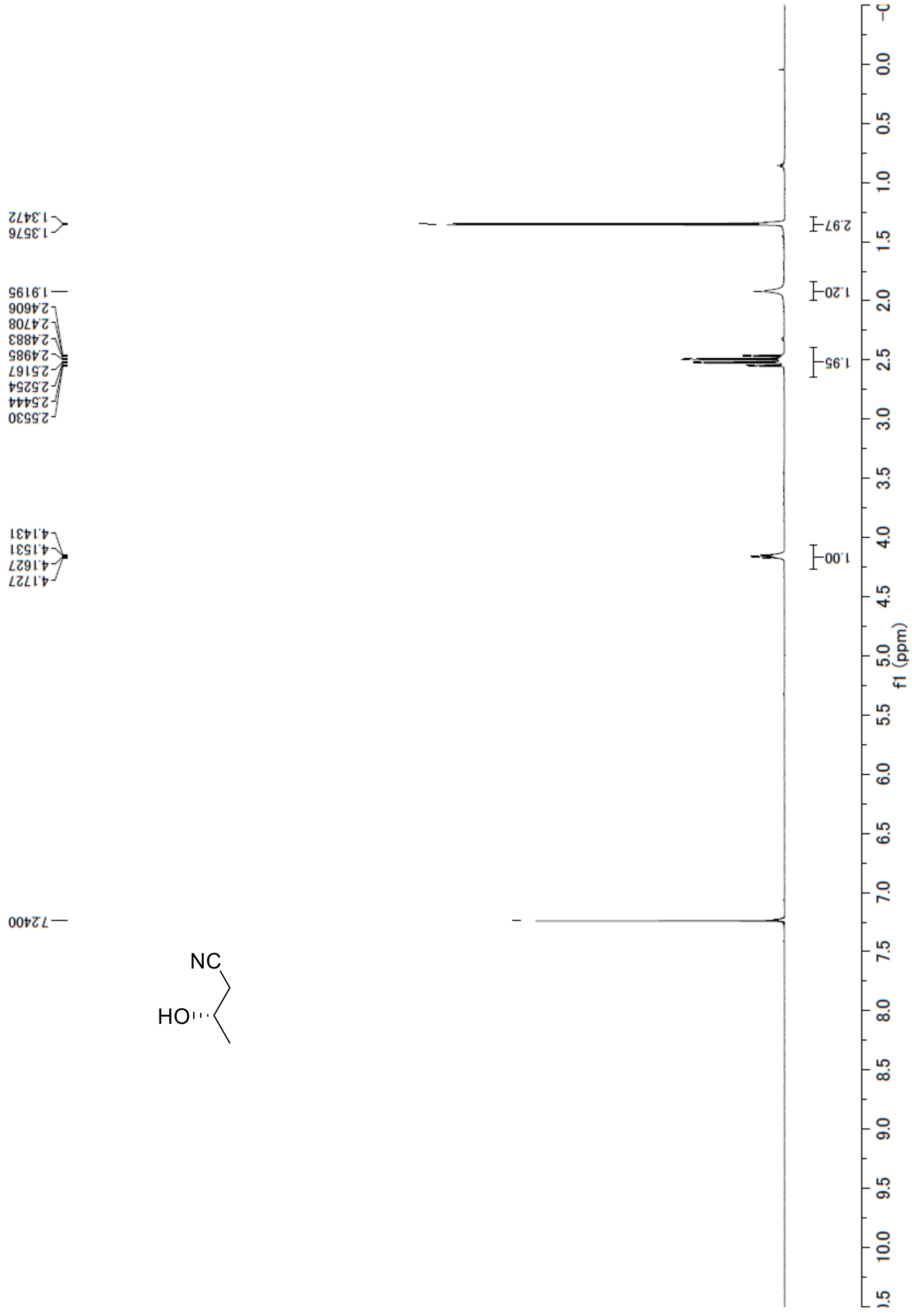

1.3472
1.3576
1.9195
2.4606
2.4708
2.4883
2.4985
2.5167
2.5254
2.5444
2.5530
4.1431
4.1531
4.1627
4.1727
7.2400
NC
HO
2.97
1.20
1.95
1.00
f1 (ppm)

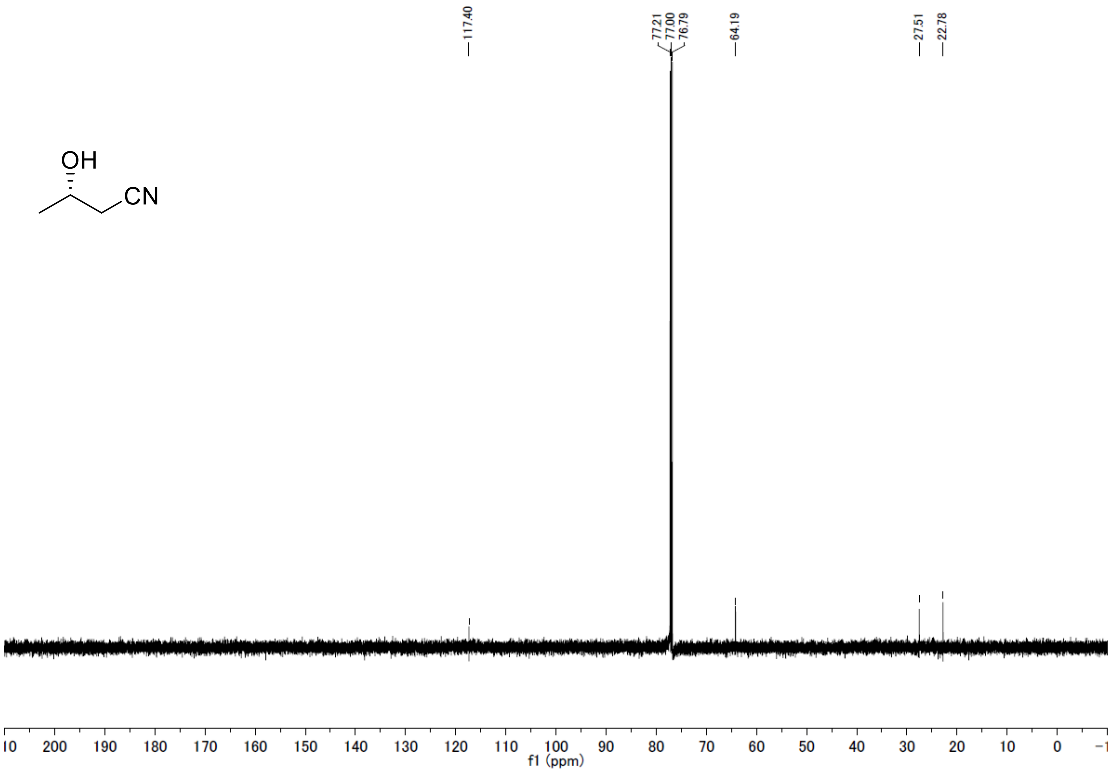

117.40
77.21
77.00
76.79
64.19
27.51
22.78
OH
CN
f1 (ppm)

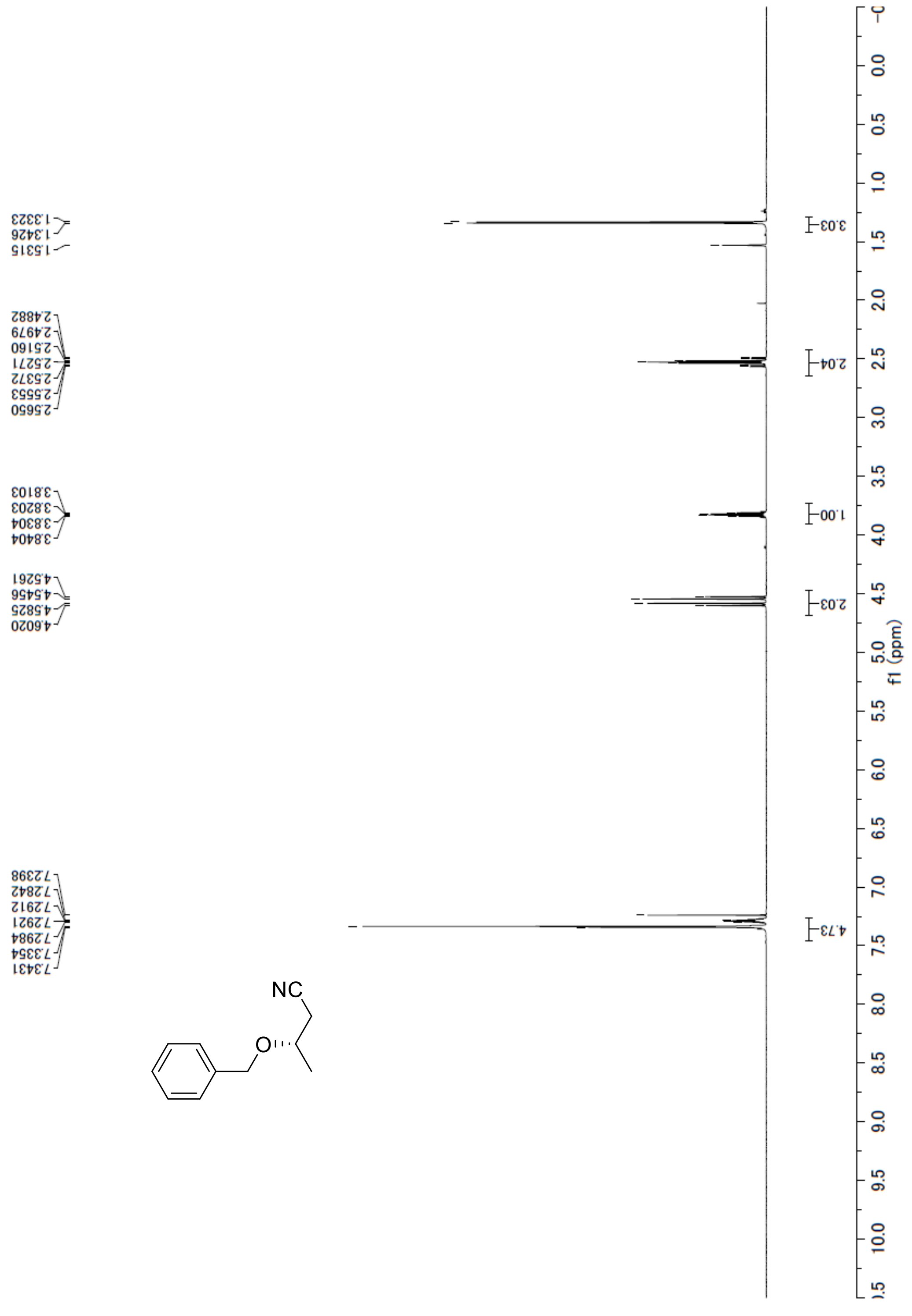
NC
O
1.3323
1.3426
1.5315
2.4882
2.4979
2.5160
2.5271
2.5372
2.5553
2.5650
3.8103
3.8203
3.8304
3.8404
4.5261
4.5456
4.5825
4.6020
7.2398
7.2842
7.2912
7.2921
7.2984
7.3354
7.3431
3.03
2.04
1.00
2.03
4.73
f1 (ppm)

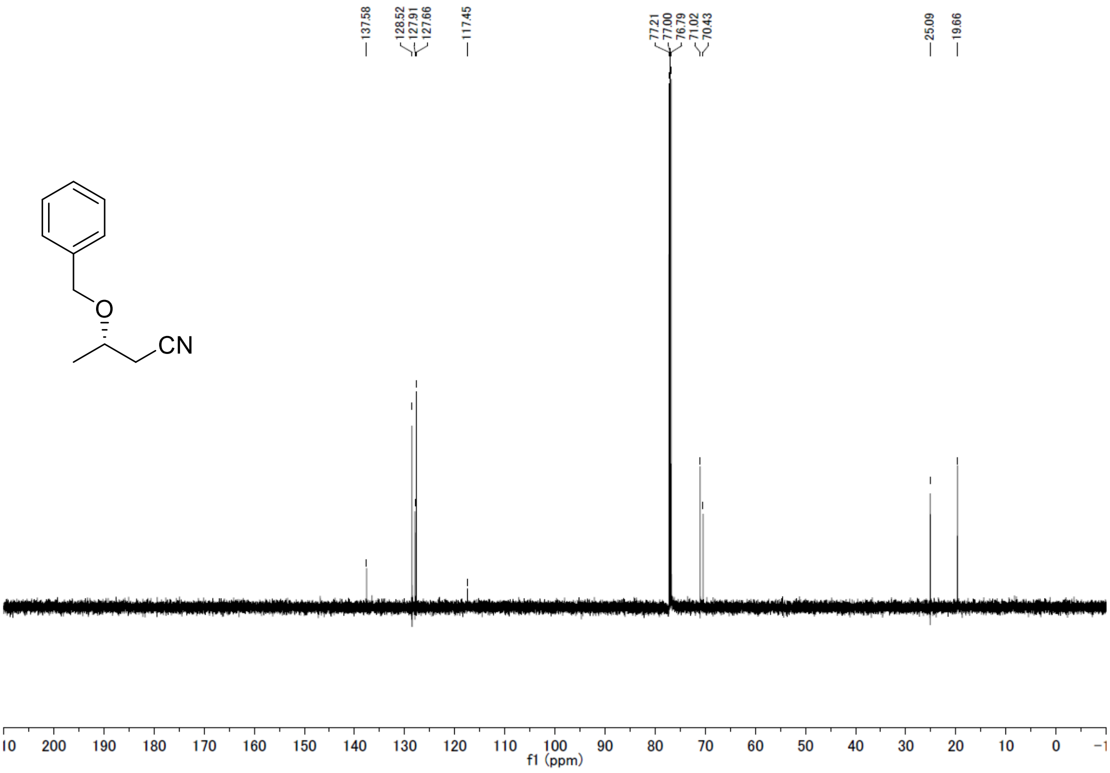

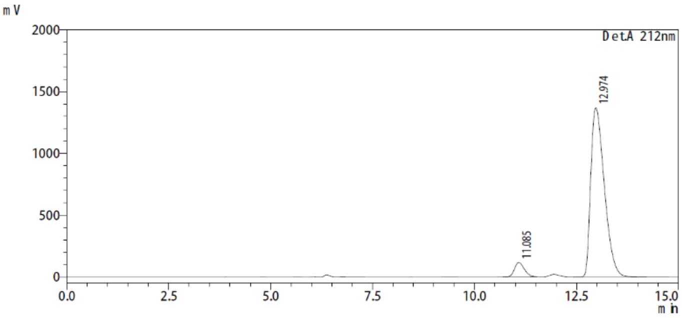

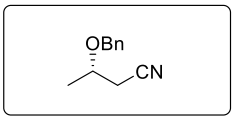

<ピークレポート>
DetA 212nm

ピーク#	保持時間	面積	高さ	濃度	単位	マーク	化合物名
1	11.085	1929061	116360	5.767		M	
2	12.974	31521471	1370076	94.233		M	
Total		33450532	1486435				

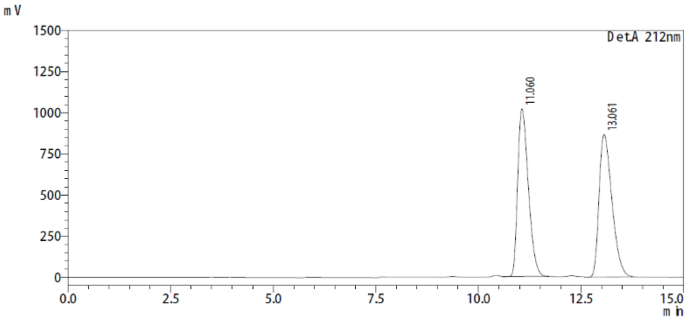

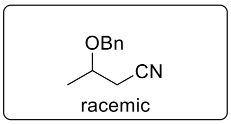

<ピークレポート>
DetA 212nm

ピーク#	保持時間	面積	高さ	濃度	単位	マーク	化合物名
1	11.060	18350465	1016681	49.299		M	
2	13.061	18872019	865725	50.701		M	
Total		37222484	1882406				

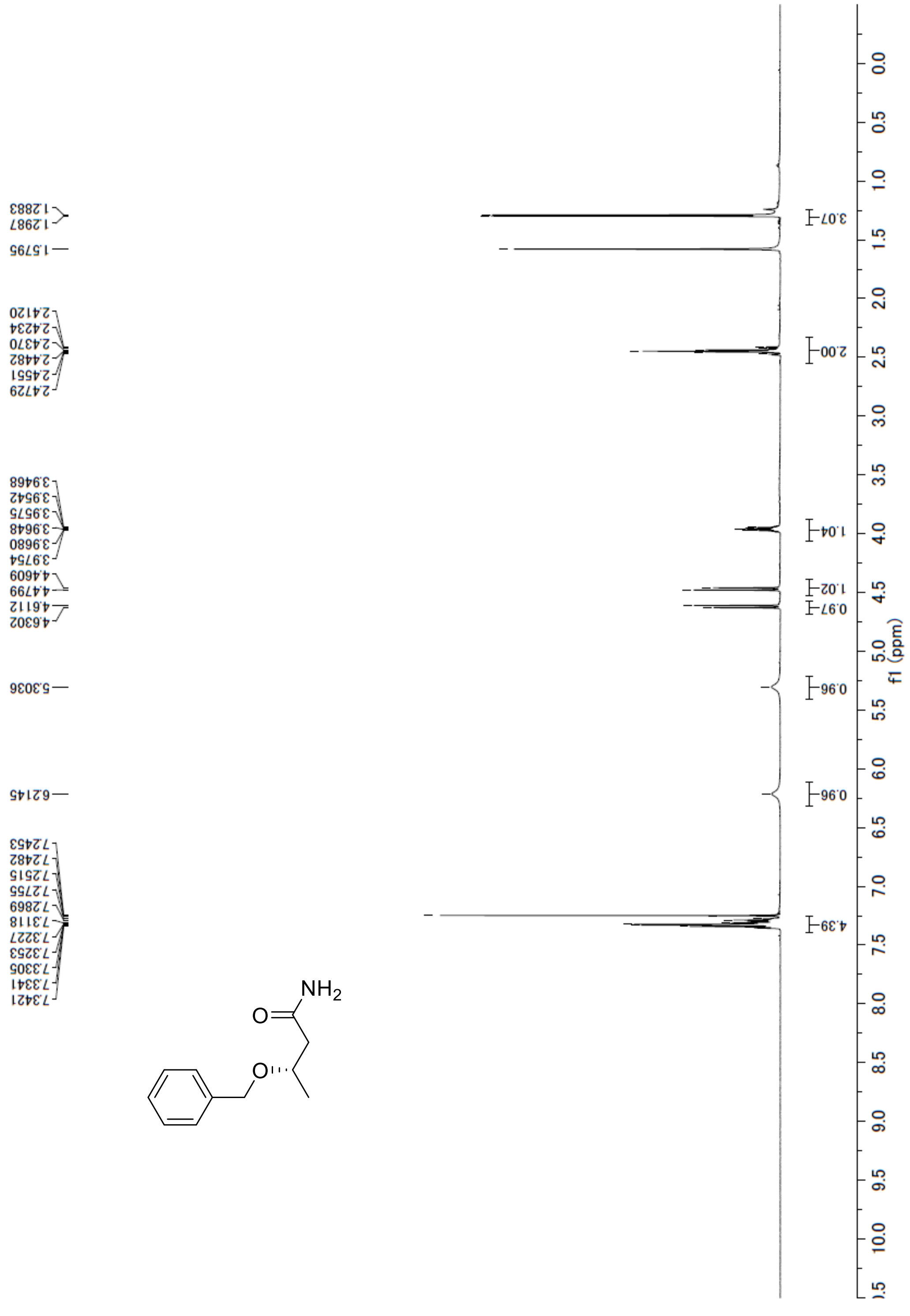
1.2883
1.2987
1.5795
2.4120
2.4234
2.4370
2.4482
2.4551
2.4729
3.9468
3.9542
3.9575
3.9648
3.9680
3.9754
4.4609
4.4799
4.6112
4.6302
5.3036
6.2145
7.2453
7.2482
7.2515
7.2755
7.2869
7.3118
7.3227
7.3253
7.3305
7.3341
7.3421
3.07
2.00
1.04
1.02
0.97
0.96
0.96
4.39
f1 (ppm)
NH2
O

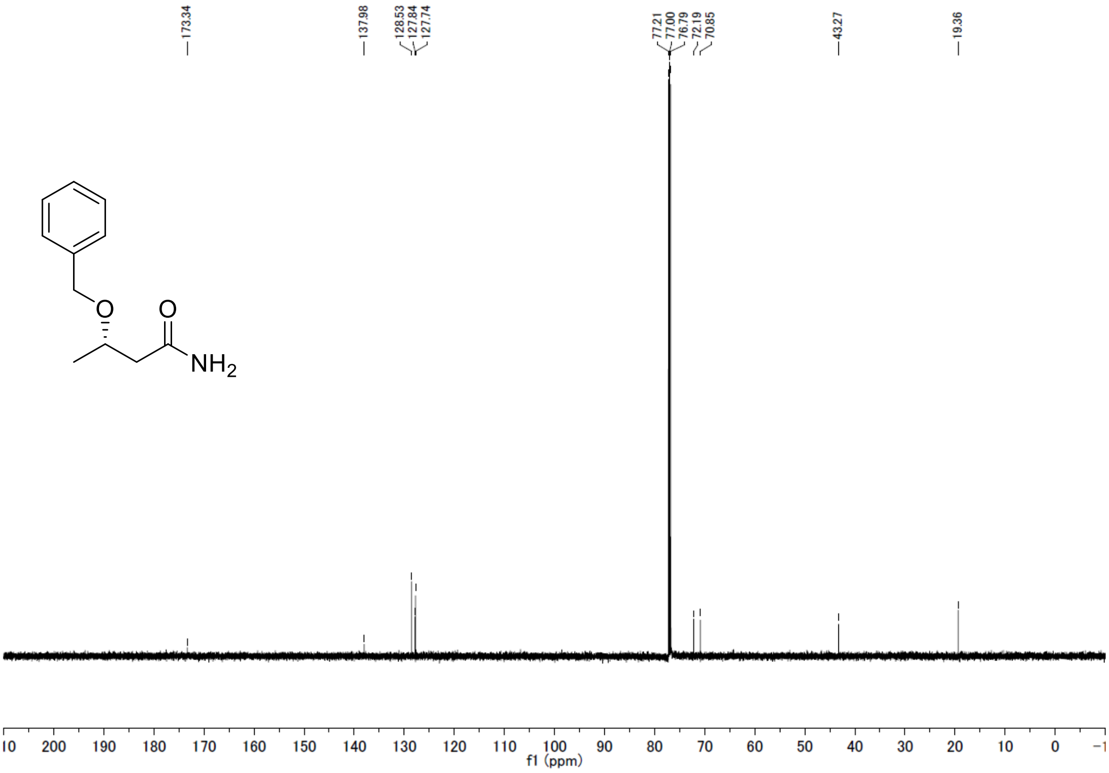

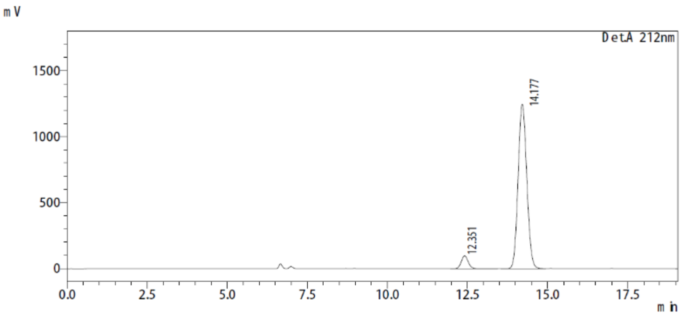

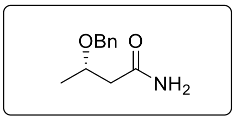

<ピークレポート>

DetA 212nm

ピーク#	保持時間	面積	高さ	濃度	単位	マーク	化合物名
1	12.351	1416682	96593	6.810		S	
2	14.177	19385681	1142550	93.190			
Total		20802363	1239143				

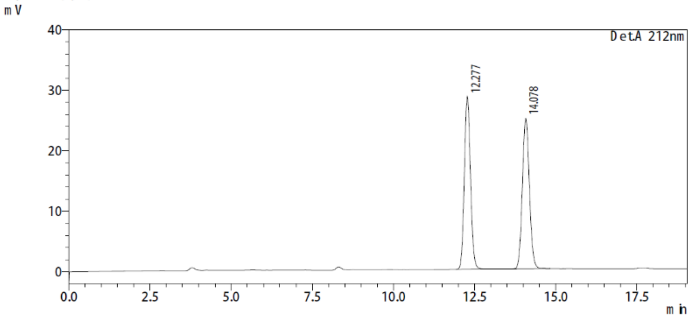

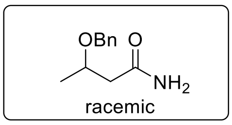

<ピークレポート>

DetA 212nm

ピーク#	保持時間	面積	高さ	濃度	単位	マーク	化合物名
1	12.277	374909	28578	49.925			
2	14.078	376042	24881	50.075			
Total		750951	53458				
